# Macrophage-Driven Biomaterial Degradation Depends on Scaffold Microarchitecture

**DOI:** 10.3389/fbioe.2019.00087

**Published:** 2019-04-26

**Authors:** Tamar B. Wissing, Valentina Bonito, Eline E. van Haaften, Marina van Doeselaar, Marieke M. C. P. Brugmans, Henk M. Janssen, Carlijn V. C. Bouten, Anthal I. P. M. Smits

**Affiliations:** ^1^Department of Biomedical Engineering, Eindhoven University of Technology, Eindhoven, Netherlands; ^2^Institute for Complex Molecular Systems (ICMS), Eindhoven University of Technology, Eindhoven, Netherlands; ^3^Xeltis B.V., Eindhoven, Netherlands; ^4^SyMO-Chem B.V., Eindhoven, Netherlands

**Keywords:** *in situ* tissue engineering, enzymatic degradation, oxidative degradation, reactive oxygen species, electrospinning, macrophage polarization, immunomodulation, foreign body response

## Abstract

*In situ* tissue engineering is a technology in which non-cellular biomaterial scaffolds are implanted in order to induce local regeneration of replaced or damaged tissues. Degradable synthetic electrospun scaffolds are a versatile and promising class of biomaterials for various *in situ* tissue engineering applications, such as cardiovascular replacements. Functional *in situ* tissue regeneration depends on the balance between endogenous neo-tissue formation and scaffold degradation. Both these processes are driven by macrophages. Upon invasion into a scaffold, macrophages secrete reactive oxygen species (ROS) and hydrolytic enzymes, contributing to oxidative and enzymatic biomaterial degradation, respectively. This study aims to elucidate the effect of scaffold microarchitecture, i.e., μm-range fiber diameter and fiber alignment, on early macrophage-driven scaffold degradation. Electrospun poly-ε-caprolactone-bisurea (PCL-BU) scaffolds with either 2 or 6 μm (Ø) isotropic or anisotropic fibers were seeded with THP-1 derived human macrophages and cultured *in vitro* for 4 or 8 days. Our results revealed that macroph age-induced oxidative degradation in particular was dependent on scaffold microarchitecture, with the highest level of ROS-induced lipid peroxidation, NADPH oxidase gene expression and degradation in the 6 μm Ø anisotropic group. Whereas, biochemically polarized macrophages demonstrated a phenotype-specific degradative potential, the observed differences in macrophage degradative potential instigated by the scaffold microarchitecture could not be attributed to either distinct M1 or M2 polarization. This suggests that the scaffold microarchitecture uniquely affects macrophage-driven degradation. These findings emphasize the importance of considering the scaffold microarchitecture in the design of scaffolds for *in situ* tissue engineering applications and the tailoring of degradation kinetics thereof.

## Introduction

The use of electrospun degradable synthetic scaffolds is being explored for the repair or replacement of various load-bearing tissues (e.g., heart valve replacement, pelvic floor reconstruction; Kluin et al., 2017; Hympánová et al., 2018). Such scaffolds are designed with the aim to induce endogenous regeneration of the replaced tissue, directly in its functional site, a process also known as *in situ* tissue engineering. Key to the success of this approach is the modulation of the scaffold-induced immune response and the utilization of the host regenerative potential. It is hypothesized that shortly after implantation the scaffold triggers a phased wound healing process, which consists of the early infiltration of immune cells followed by the attraction of tissue producing cells, the secretion of extracellular matrix (ECM) components, and, ultimately, the regeneration of a functional, organized native-like tissue (Wissing et al., 2017). Importantly, over time, the scaffold should degrade in order to avoid chronic inflammation and scar tissue formation. The loss of structural integrity and mechanical properties occurring during degradation should be promptly compensated for by the presence of newly formed tissue. Therefore, the degradation kinetics of the implanted electrospun biomaterials represent a critical parameter for successful *in situ* tissue engineering.

Even though the exact mechanism behind degradation of synthetic materials *in vivo* remains poorly understood, various groups have linked it to the immune cells infiltrating the scaffolds and, particularly, to phagocytes, e.g., neutrophils and macrophages (Anderson et al., 2008; Generali et al., 2014). Upon biomaterial implantation, phagocytes adhere to the scaffold and synthesize large amounts of degradative products, such as hydrolytic enzymes, like lysosomal acid lipase (LIPA) and cholesterol esterase, and/or reactive oxygen species (ROS), a process mediated by the nicotinamide adenine dinucleotise phosphate (NADPH) oxidase-2 complex (Pastorino et al., 2004; McBane et al., 2007; Brown and Griendling, 2009; Martins et al., 2009; Peng et al., 2010; Brugmans et al., 2015) While neutrophils govern the initial acute inflammatory response, macrophages quickly become the predominant cell type and remain present at the biomaterial interface until the degradation process is finalized (Anderson, 1993; Labow et al., 2001a). In the presence of large scaffold remnants, macrophages tend to fuse to form foreign body giant cells (FBGCs) and undertake frustrated phagocytosis. Ultimately, FBGCs release large quantities of ROS, degradative enzymes and acids in the ultimate attempt to break down the scaffold (Anderson et al., 2008).

Previously, it was shown that scaffold microarchitecture profoundly influences macrophage adhesion, infiltration and differentiation into the classical pro-inflammatory phenotype (M1) and the alternative pro-regenerative phenotypes (e.g., M2a and M2c; Balguid et al., 2009; Kurpinski et al., 2010; Saino et al., 2011; Garg et al., 2013; McWhorter et al., 2013, 2015; Wang et al., 2014; Wissing et al., 2017). More specifically, increasing fiber diameter in the μm range positively correlated with the expression of M2 markers *in vitro* (Garg et al., 2013; Wang et al., 2014), and improved regenerative outcomes *in vivo* (Wang et al., 2014). Although the exact mechanisms behind such microarchitecture-induced variations in macrophage polarization state are unclear, they may be caused by modulation of cellular morphology. Induction of a more elongated spindle-shaped morphology using 2D micropatterned substrates *in vitro* was proposed to stimulate alternative macrophage polarization (M2a) following a distinct actin-related pathway, independent of the biochemical environment (McWhorter et al., 2013). Correspondingly, fiber alignment was shown to minimize the host response, enhancing scaffold-tissue integration while minimizing fibrous capsule formation *in vivo* (Cao et al., 2010).

To date, little effort has been undertaken in unraveling the influence of scaffold microarchitecture on macrophage-driven degradation and, importantly, in the possible relationship between macrophage specific phenotypes and their capability to degrade synthetic scaffolds. Therefore, our goal was to elucidate the influence of scaffold microarchitecture (i.e., fiber diameter in the μm range and fiber alignment) on early macrophage-driven enzymatic and oxidative degradation (up till 8 days). We hypothesized that isotropic scaffolds with a smaller fiber diameter would promote pro-inflammatory macrophage polarization, and consequently, stimulate the production of hydrolytic enzymes and ROS, thereby significantly accelerating scaffold degradation. This hypothesis was tested by direct seeding of THP-1 derived human macrophages onto electrospun scaffolds with two different fiber diameters (i.e., fiber Ø of 2 and 6 μm) and alignments (isotropic or anisotropic). To relate possible differences in macrophage degradative behavior to scaffold-induced macrophage polarization, we investigated the polarization profiles of the macrophages cultured on the different scaffold microarchitectures, and compared the phenotypical profiles to those macrophages seeded in 6 μm isotropic scaffolds with the exogenous addition of cytokines to induce polarization into the M1, M2a, and M2c macrophage phenotypes.

For this study, we have selected bis-urea-modified poly-ε-caprolactone (PCL-BU) as the scaffold material (Wisse et al., 2006). The bis-urea units in this synthetic biomaterial interact supramolecularly through hydrogen bonds, giving rise to its beneficial elastomeric properties. In general, supramolecular biomaterials show a relative ease of processing and provide the possibility to finely tune the morphological, chemical, and thereby, degradation properties (Bouten et al., 2011; Brugmans et al., 2015; van Almen et al., 2016). Accordingly, these materials are being tested with regard to *in situ* tissue engineering for cardiovascular applications (Muylaert et al., 2015; van Almen et al., 2016; Bockeria et al., 2017; Kluin et al., 2017; Bennink et al., 2018). PCL-BU scaffolds, specifically, have been tested as vascular grafts, revealing relatively rapid degradation kinetics *in vivo* (Duijvelshoff et al., 2018). Accelerated *in vitro* degradation studies have shown that PCL-BU is susceptible to oxidative degradation and, to a lesser extent, to enzymatic degradation, resulting in fiber cleavage and surface erosion of the electrospun scaffold fibers (Brugmans et al., 2015).

## Materials and Methods

### Experimental Outline

**Figure 1:**
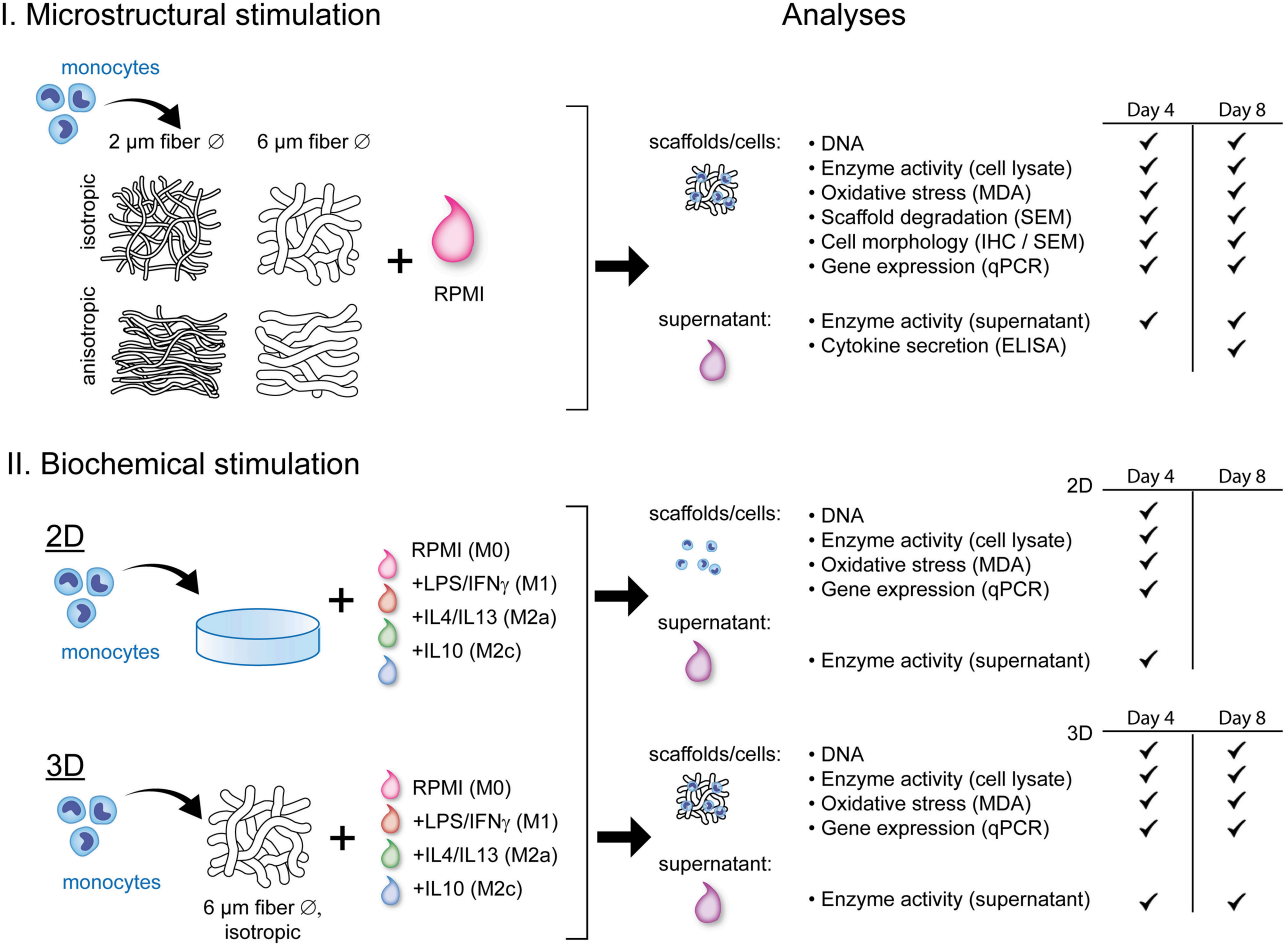
Schematic representation of the experimental outline and analyses. THP-1 derived macrophages were exposed to either scaffold microstructural stimuli **(I)** or biochemical stimuli **(II)** and analyzed for their degradation profile and phenotype. RPMI, Roswell Park Memorial Institute; 1640 medium; LPS, lipopolysaccharide; IFN-γ, interferon gamma; IL, interleukin; MDA, malondialdehyde; SEM, scanning electron microscopy; IHC, immunohistochemistry; ELISA, enzyme-linked immunosorbent assay; qPCR, quantitative polymerase chain reaction.

Figure 1 displays a schematic overview of the experimental setup and the corresponding analyses. To study the effect of scaffold microarchitecture on macrophage-driven early scaffold degradation and macrophage phenotype, THP-1 derived human macrophages were seeded onto scaffolds with varying microarchitectures. After 4 and 8 days, the cell-seeded scaffolds and the supernatant were collected for analysis. To test the effect of biochemically induced macrophage polarization on the cell phenotype and degradative profile, macrophages were cultured in 2D cell culture plates or 3D scaffolds (6 μm Ø, isotropic), and supplemented with cytokines to induce macrophage polarization into the M1, M2a, and M2c subtypes. Samples and supernatant were collected at day 4 (2D) or day 4 and 8 (3D) for analysis.

### Scaffold Preparation and Sterilization

PCL-BU was supplied by SyMO-Chem (Eindhoven, the Netherlands) and processed into electrospun fibrous scaffolds with different fiber diameters and orientations in a climate controlled electrospinning cabinet (EC-CLI, IME Technologies, Geldrop, the Netherlands). The fibrous meshes were produced from polymer solutions containing either 10% (w/w) or 15% (w/w) of PCL-BU in chloroform (CHCl_3_; amylene-stabilized, Sigma Aldrich, 372978). Solutions were dispensed through a charged nozzle and collected on a rotating cylindrical mandrel (Ø 35 mm) while keeping temperature (25°C) and relative humidity (30%) constant. The various microarchitectures were produced according to the settings of Table 1. After removal from the mandrel, scaffolds were placed under vacuum overnight to remove solvent remnants. From the rectangular (10 × 10 cm^2^) meshes with various microarchitectures, Ø 8 mm circular scaffolds were punched (biopsy punch, Kai Medical, Japan), placed in 10 mL of sterile Phosphate Buffered Saline (PBS, Sigma Aldrich, P4417) and centrifuged for 10 min at 4,500 rpm to wash and increase hydrophilicity. Both sides of the scaffolds were sterilized via exposure to UV light for 15 min.

**Table 1:** Electrospinning settings for the different scaffold microarchitectures.

**Fiber Ø [μm]**	**Alignment**	**Flow rate [μL]**	**Nozzle-to-mandrel [cm]**	**Nozzle voltage [kV]**	**Mandrel voltage [kV]**	**Rotation speed [rpm]**
2	Isotropic	10	24	20	−2	100
	Anisotropic	10	24	20	−2	1,600
6	Isotropic	40	16	20	−1	100
	Anisotropic	40	16	20	−1	2,300

### Scaffold Microarchitecture and Mechanical Properties

Scaffold microarchitectures were visualized with scanning electron microscopy (SEM, Quanta 600F; Thermo Fisher, Hillsboro, OR) to assess fiber morphology, diameter, and distribution. Average fiber diameter, pore area (μm^2^) and geometry (average width/height ratio) per group were determined manually by 30 individual measurements of randomly chosen fibers/pores in three representative images per mesh using the Image J software (U.S. National Institutes of Health, Bethesda, MD, USA). Fiber distribution and orientations were quantified via custom Matlab scripts, as described elsewhere (van Haaften et al., 2018). The surface to volume ratio was calculated using the following equations (Boland et al., 2005):

$$\begin{array}{l} \text{s}urface\;to\;volume\;rati\text{o}=\frac{2\pi {\text{r}}_{\text{f}}\text{L}}{\text{Vs}}\\ \;\text{L}=\frac{\text{Ms}}{\rho_{\text{s}}\pi {\text{r}}_{\text{f}}^{2}}\\ \;\text{Vs}=\frac{\text{Ms}}{\rho_{\text{s}}} \end{array}$$

where *r*_*f*_ is the average radius of the fiber, *L* is the fiber length, *Vs* is the volume of the scaffold, *Ms* is the mass of the scaffold and ρ_*s*_ is the density of the material.

The mechanical properties of each mesh were characterized using a biaxial tensile tester (CellScale Biomaterial Testing, Waterloo, Canada) equipped with a 5 N load cell. From each mesh, 5 × 5 mm^2^ samples were cut and aligned along the actuator directions (x = parallel to electrospinning direction; y = perpendicular to electrospinning direction). After mounting, graphite particles were sprayed on the sample's surface for optical strain analysis. The samples were then equibiaxially stretched up to 20% strain at a 100%/min strain rate. Scaffold thicknesses were optically measured at 10 locations using bright field microscopy (Keyence VHX-500FE). All tests were performed at room temperature in dry conditions. Cauchy stress-stretch curves were obtained based on the force and displacements measurements.

### Cell Expansion

The human monocytic cell line THP1 (Sigma Aldrich, lot# 16K052) was thawed and expanded in Roswell Park Memorial Institute (RPMI) 1640 medium (A10491-0, Gibco), supplemented with fetal bovine serum (10% v/v; FBS, Bovogen) and penicillin/streptomycin (1% v/v; P/S, Lonza, Basel, Switzerland, DE17-602E) (full medium) in a standard cell culture incubator. Medium was replenished every 2–3 days to maintain cell densities between 0.5–1.0 × 10^6^ cells/ml of medium. Cells were seeded at passage 14.

### Cell Seeding and Monocyte-to-Macrophage Differentiation

Punched scaffolds were clamped between a custom-made polyether ether ketone (PEEK) ring and a membrane-free Trans-well insert (Corning, Sigma Aldrich, CLS3472), and UV sterilized (10 min/scaffold side). Scaffolds were incubated overnight in RPMI medium supplemented with 10% FBS and 1% P/S to allow for protein adsorption. Prior to cell seeding, medium was removed and 300 μL of cell suspension containing 3 × 10^6^ cells and 50 ng/ml phorbol 12-myristate 13-acetate (PMA, Sigma Aldrich, P8139) was pipetted on top of each scaffold. Cell-seeded scaffolds were centrifuged at 150 g (10 min at 21°C) to promote cell infiltration, and subsequently transferred to new 24-well cell culture plates (Corning Costar, CLS3738) containing PMA enriched medium (50 ng/ml). The well-plates were incubated for 2 days to stimulate monocyte-to-macrophage differentiation. For the 2D control cultures, monocytes were seeded at a density of 1.5 × 10^5^/cm^2^ in polystyrene 6-well cell culture plates (Greiner Bio-one, 657160) and stimulated with PMA enriched medium (50 ng/ml) for 2 days. After 2 days, the PMA-enriched medium was removed and replaced with RPMI medium supplemented with 10% FBS and 1% P/S for 24 h (Lund et al., 2016). Macrophages were kept in culture for an additional 4 (2D and 3D) or 8 (3D) days and medium was replenished every 2 days. Culture media (day 4 and 8) were collected, centrifuged (300 G, 5 min, 4°C) and the supernatants were stored at −80°C until further use. At sacrifice, samples (in 2D and 3D) were either washed in sterile PBS (2 times 5 s) and directly analyzed, or snap frozen in liquid nitrogen and stored at −80°C until further analysis.

### Macrophage Polarization

As control groups, cells in 2D (seeded on tissue culture treated polystyrene) and 3D (isotropic 6 μm fiber diameter PCL-BU scaffolds) were biochemically stimulated toward the three different lineages of the macrophage spectrum, namely the pro-inflammatory M1 macrophages, the anti-inflammatory M2a macrophages, and the regulatory M2c macrophages (Mosser and Edwards, 2009; Sica and Mantovani, 2012). Particularly, the pro-inflammatory M1 group was exposed to medium supplemented with 100 ng/mL of lipopolysaccharide (LPS, Sigma Aldrich, L2880) and 20 ng/mL interferon gamma (IFNγ, Peprotech, 300-02) (Fearing and Van Dyke, 2014). The other two groups received either 20 ng/mL interleukin-4 (IL-4, Peprotech, 200-04) and interleukin-13 (IL-13, Peprotech, 200-13) or 50 ng/mL interleukin-10 (IL-10, Peprotech, 200-10) to induce M2a or M2c polarization, respectively (Ambarus et al., 2012; Freytes et al., 2013). Cytokine-enriched media were refreshed every 2 days and, at day 4 or day 8, collected and stored at −80°C until further analysis. Samples were sacrificed as described in section Cell Seeding and Monocyte-to-Macrophage Differentiation.

### Analyses

Each scaffold was divided into four equal quarters of which one quarter was used for DNA quantification, scanning electron microscopy (SEM) evaluation or immunostainings. The other three quarters were used either for enzymatic activity determination, oxidative stress quantification or RNA extraction. Supernatants were collected and used for enzyme activity and protein secretion determination (ELISA).

#### DNA Quantification

For DNA quantification, scaffold quarters (*N* ≥ 8/group/time point) were disrupted with a microdismembrator (Sartorius, Goettingen, Germany) 2 times for 30 s at 3,000 rpm, using Ø 3 mm beads (Sartorius, Goettingen, Germany) in Nalgene cryogenic vials (Thermo Scientific, 5000-0012). After disruption, 500 μL of papain-enriched digestion buffer (100 mM phosphate buffer (pH = 6.5), 5 mM L-cysteine (C-1276), 5 mM ethylene-di-amine-tetra-acetic acid (EDTA, ED2SS), and 140 μg of papain (P4762) per mL, all from Sigma Aldrich) was added, mixed, transferred to a new Eppendorf tube and incubated at 60°C for 16 hrs for digestion.

For the 2D cell culture samples (*n* = 9/group/time point), 400 μL/well of ultra-pure H_2_O was added to facilitate cell scraping. Cells were scraped on ice and the cell solution was transferred to a new Eppendorf tube, snap frozen in liquid nitrogen and stored at −80°C until further analysis. Prior to digestion, samples were thawed, supplemented with 500 μL of papain digestion buffer and incubated at 60°C for 16 hrs.

After digestion, samples were vortexed and centrifuged at 12,000 rpm for 10 min. The supernatant was used for DNA quantification using a Qubit dsDNA BR assay kit (Life Technologies, Carlsbad, California, USA) and a Qubit fluorometer (Life Technologies) according to the manufacturer's protocol.

#### Enzymatic Activity Quantification

The supernatant and cell lysate from the 2D and 3D cell-cultures collected at day 4 and 8 were assayed for enzyme activity (*N* ≥ 4/group/time point) via enzyme, predominantly esterase induced p-nitrophenyl butyrate (pNPB, Sigma Aldrich, N9876) to yellow p-nitrophenol (pNP) conversion, which was measured by UV absorbance at 405 nm (Moore et al., 1996; Labow et al., 2001a). To obtain the cell lysate, three-quarters of the scaffold were disrupted as described in section DNA Quantification, and resuspended in 1 mL of Triton-X solution (0.05% in PBS). The solution was transferred to a new Eppendorf tube, snap frozen in liquid nitrogen and stored at −80°C until further analysis. Before analysis, supernatant and lysate samples were thawed, vortexed and centrifuged at 12,000 rpm, for 5 min. One hundred fifty microliter of the supernatant or cell lysate was added to a 96-well plate (Greiner bio-one, 655101) together with 140 μL of Tris buffer (1 M, 7.5 pH, Merck, 1.08382.0500) and 10 μL of pNPB solution [3.55 μL pNPB in 5.0 ml of acetonitrile (Sigma Aldrich, 271004)]. UV absorbance at 37°C was determined every 5 min via a microplate reader (Synergy HTX multimode-reader, Biotek). A dilution series of cholesterol esterase (Sigma Aldrich, C3766) in full RPMI medium or 0.05% Triton-X solution (Merck, 1.08603.100) were included as positive controls. Full RPMI medium and 0.05% Triton-X solution were used as negative controls. All samples were measured in duplicate. Enzymatic activity was expressed in units (1 unit = 1 nmol pNP released from pNPB per minute at 37°C) and corrected for the amount of DNA measured for each experimental condition.

#### Oxidative Stress Quantification

The level of malondialdehyde (MDA), a by-product of oxidative stress induced lipid peroxidation (MDA assay kit, Sigma Aldrich, MAK085) was quantified per well (2D) and on three-quarters of the scaffold (*N* = 6/group/time point) as an indirect measure for ROS production and oxidative damage. Three hundred microliter of MDA assay specific lysis buffer was added to the wells (2D) or to the disrupted scaffolds. After 5 min of incubation on ice, lysis buffers were transferred to new Eppendorf tubes and centrifuged for 10 min at 11.000 rpm to remove insoluble material. Two hundred microliter of the supernatant was mixed with 600 μL of thiobarbituric acid (TBA) to form a fluorometric product, which is proportional to the MDA amount. Fluorometric products were quantified using a microplate reader (Synergy HTX multimode-reader, Biotek), with an excitation/emission wavelength of 532/553 nm. All values were normalized for DNA content.

#### Gene Expression: qPCR

Snap frozen scaffolds (*N* ≥ 6/group / time point) were disrupted as described in the paragraph DNA Quantification and lysed in RLT buffer containing β-mercaptoethanol (Sigma Aldrich, M3148). 2D cell cultures (*N* = 6/group/time point) were directly lysed in the well-plates on ice. A Qiagen RNeasy kit was used for RNA isolation according to supplier instructions and a DNAse incubation step (Qiagen, 74106) of 30 min was included to remove genomic DNA contamination. A spectrophotometer (NanoDrop, ND-1000, Isogen Life Science, The Netherlands) was used to determine RNA quantity and purity. RNA integrity was checked via gel electrophoresis. cDNA was synthesized starting from 100 or 200 ng of RNA in a 20 μL reaction solution that consisted of RNAse-free ultra-pure water (ddH_2_O), 1 μL of random primers (50 ng/μl, Promega, C1181), 1 μL of dNTPs (10 mM, Invitrogen), 4 μL of 5x first strand buffer, 2 μL of 0.1 M DTT and 1 μL of M-MLV Reverse Transcriptase (RT) (200 U/μl, Invitrogen, 28025-013, Breda, the Netherlands). cDNA synthesis was performed in a thermal cycler (C1000 Touch, Bio-Rad) via the following cycle: 65°C (5 min), on ice (2 min) while adding the enzyme mixture, 37°C (2 min), 25°C (1 min), 37°C (50 min), and 70°C (15 min). Absence of genomic contamination was investigated using conventional PCR with glyceraldehydes-3-phosphate dehydrogenase (GAPDH) primers, and gel electrophoresis. qPCR was performed using the primer sequences listed in Tables 2, **4**, and Supplementary Table S1. The selected primers are coding for proteins related to cell phenotype, tissue deposition, remodeling and cell fusion (Table 3). GAPDH and CYC-1 were selected as reference genes. Gene expression was determined by adding 1,000 nM primer mix (GAPDH,CYC-1, MMP-9) or 500 nM primer mix (others primers), 5 μl of SYBR Green Supermix (Bio-Rad, 170-8886) and an additional 1.5 or 1.75 μl of ddH_2_O to all the cDNA samples (3 μl of diluted cDNA). Real-time PCR (CFX384, Bio-rad) was performed via the following thermal protocol: 95°C for 3 min, a cycle of each 95°C for 20 s, 60°C for 20 s, and 72°C for 30 s repeated 40 times, 95°C for 1 min, 65°C for 1 min and finally a melting curve measurement. C_t_ values were normalized for the reference genes and relative expression levels were calculated via the 2^−ΔCt^ formula.

**Table 2:** Primers for gene expression analysis.

**Primer**	**Symbol**	**Accession number**	**Primer sequence (5′-3′)**
Monocyte chemoattractant protein 1	MCP1	NM_002982	FW: CAGCCAGATGCAATCAATGCC
			RV: TGGAATCCTGAACCCACTTCT
Cluster of differentiation 68	CD68	NM_001040059.1	FW: CTACTGGCAGAGAGCACTGG
			RV: CCGCCATGTAGCTCAGGTAG
Chemokine (C-C motif) receptor 7	CCR7	NM_001838	FW: AAGCCTGGTTCCTCCCTATC
			RV: ATGGTCTTGAGCCTCTTGAAATA
Tumor necrosis factor alpha	TNF	NM_000594	FW: GAGGCCAAGCCCTGGTATG
			RV: CGGGCCGATTGATCTCAGC
Interleukin 6	IL6	NM_000600	FW: ACTCACCTCTTCAGAACGAATTG
			RV: GTCGAGGATGTACCGAATTTGT
Cluster of differentiation 200 cell surface glycoprotein receptor	CD200R1	NM_170780	FW: GAGCAATGGCACAGTGACTGTT
			RV: GTGGCAGGTCACGGTAGACA
Mannose receptor c, type 1	CD206 (MRC-1)	NM_002438	FW: TGGGTTCCTCTCTGGTTTCC
			RV: CAACATTTCTGAACAATCCTATCCA
Cluster of differentiation 163	CD163	NM_004244	FW:CACTATGAAGAAGCCAAAATTACCT
			RV: AGAGAGAAGTCCGAATCACAGA
Interleukin 10	IL10	NM_000572	FW: GACTTTAAGGGTTACCTGGGTTG
			RV: TCACATGCGCCTTGATGTCTG
Transforming growth factor, beta 1	TGFB1	NM_000660	FW: GCAACAATTCCTGGCGATACCTC
			RV: AGTTCTTCTCCGTGGAGCTGAAG
Matrix metalloproteinase 9	MMP9	NM_004994	FW: TGGGGGGCAACTCGGC
			RV: GGAATGATCTAAGCCCAG
Nicotinamide adenine dinucleotide phosphate-oxidase 2	NOX2	NM_000397.3	FW: AACTGGGCTGTGAATGAGGG
			RV: GCCAGTGCTGACCCAAGAA
Nuclear factor kappa-light-chain-enhancer of activated B cells	NFκB	NM_001165412	FW: AGACCAAGGAGATGGACCTCA
			RV: GCATTGGGGGCTTTACTGTC
Lipase A or cholesterol ester hydrolase	LIPA	NM_001288979.1	FW: TCCTGCTGGAACTTCTGTGC
			RV: ACTGCTTCCCCAGTCAAAGG
Cluster of differentiation 44	CD44	NM_000610.3	FW: TCAGCAAGAATTTGATCGTTCCAG
			RV: TTAGAAGCCATCCATAGCACACC
Cluster of differentiation 47	CD47	NM_001777.3	FW: TGCATGGCCCTCTTCTGATT
			RV: AGGGGTTCCTCTACAGCTT
Signal regulatory protein alpha	SIRPA	NM_001040022.1	FW: TCAAATACCGCCGCTGAGAA
			RV: TGTGATATCATTTGTGTCCTGTGT
Purinergic receptor P2X 7	P2RX7	NM_002562.5	FW: ACAGTGTCTTTGACACCGCA
			RV: CCAGGCAGAGACTTCACAGG

**Table 3:** Genes and proteins analyzed via qPCR and Multiplex ELISA.

**Protein**	**Symbol**	**Function**	**qPCR**	**ELISA**
Cluster of differentiation 68	CD68	Pan-macrophage marker	x	
Monocyte chemoattractant protein 1	MCP-1	Chemotactic for monocytes/macrophages	x	x
Chemokine (C-C motif) receptor 7	CCR7	Pro- inflammatory macrophage marker	x	
Interferon gamma	IFN-γ	Pro-inflammatory factor, inhibitor of collagen production and cell proliferation		x
Tumor necrosis factor alpha	TNF-α	Pro-inflammatory factor, stimulus for collagen production, inhibitor of elastogenesis	x	x
Interleukin 6	IL-6	Pro-inflammatory factor	x	x
Cell surface glycoprotein receptor 200	CD200R1	Anti-inflammatory macrophage marker	x	
Mannose receptor c, type 1	CD206 (MRC-1)	Anti-inflammatory macrophage marker	x	
Cluster of differentiation 163	CD163	Anti-inflammatory macrophage marker	x	
Interleukin 10	IL-10	Anti-inflammatory cytokine, inhibitor of collagen production	x	x
Interleukin 13	IL-13	Anti-inflammatory macrophage marker, stimulus for collagen production		x
Transforming growth factor beta 1	TGFβ_1_	Anti-inflammatory factor; stimulus for collagen formation	x	x
Matrix metalloproteinase 9	MMP-9	Anti-inflammatory factor involved in extra-cellular breakdown and remodeling	x	x
Matrix metalloproteinase 1	MMP-1	Extra-cellular breakdown and remodeling, collagenase		x
Metallopeptidase inhibitor 1	TIMP-1	Inhibitor of MMP's		x
Platelet derived growth factor- subunit BB	PDGF-BB	Stimulus for collagen formation and cell proliferation		x
Basic fibroblast growth factor	bFGF	Stimulus for collagen formation, promotes scarless healing		x
Connective tissue growth factor	CTGF	Stimulus for collagen formation		x
Elastase	ELA	Elastin breakdown		x
Nicotinamide adenine dinuc-leotide phosphate-oxidase 2	NOX2	Contributor of ROS production	x	
Nuclear factor kappa-light-chain-enhancer of activated B cells	NFκβ	Involved in cellular responses to oxidative stress and cell survival	x	
Lipase A or cholesterol ester hydrolase	LIPA	Lysosomal enzyme	x	
Cluster of differentiation 44	CD44	Cell adhesion, migration and fusion	x	
Cluster of differentiation 47	CD47	Cell adhesion, migration and fusion	x	
Signal regulatory protein alpha	SIRPA	Cell fusion and phagocytosis	x	
Purinergic receptor P2X 7	P2RX7	Regulator of caspase activity, cytokine secretion, cell apoptosis and fusion	x	

#### Protein Secretion: Multiplex ELISA

The protein secretion levels were quantified in the pooled supernatants at day 8 (from the 3D microarchitecture samples only; *N* = 3/group) at the Multiplex core facility of the Laboratory for Translational Immunology of the University Medical Centre Utrecht, the Netherlands, using a multiplex immunoassay based on Luminex technology. Briefly, samples were incubated for 1 h with antibody-conjugated MagPlex microspheres (BiorRad, Hercules, CA), followed by 1 h exposure to biotinylated antibodies and subsequent 10 min incubation with phycoerythrin-conjugated streptavidin diluted in high performance ELISA (HPE) buffer (Sanquin). Data acquisition was executed using a FLEXMAP 3D system controlled with xPONENT 4.1 software (Luminex, Austin, TX). Data was analyzed fitting a 5-parametric curve with Bio-Plex Manager software (version 6.1.1, Biorad). A panel of proteins related to macrophage polarization, ECM breakdown and remodeling and tissue deposition was analyzed (Table 3). The obtained secretion levels were corrected for the amount of DNA measured per experimental condition.

#### Cellular Morphology and Scaffold Degradation: Scanning Electron Microscopy

The 3D samples sacrificed at day 4 and day 8 were fixed in 2.5% glutaraldehyde grade I (Sigma Aldrich, G5882), rinsed in PBS (3 × 5 min), and stored in PBS at 4°C until use. Prior to analysis, scaffolds were dehydrated in an ordered ethanol (EtOH) series (from 50 to 100% EtOH) and placed in vacuum to let the EtOH evaporate. Samples were visualized with SEM in low vacuum, using an electron beam of 10 kV (Quanta 600F; Thermo Fisher, Hillsboro, OR). After SEM analysis, scaffolds were decellularized with 4.6% natrium hypochlorite for 15 min at room temperature. Samples were washed twice in water and analyzed via SEM again to determine scaffold degradation. Images were taken at 3 different locations/sample (3 magnifications/location, i.e., 100X, 500X, and 1500X). Fiber cleavage and fiber erosion were scored for each image (as absent, low, moderate, or severe) by two researchers independently (see Supplementary Figure S1).

#### Cellular Infiltration and Actin Cytoskeleton: Stainings and Microscopy

Scaffolds sacrificed at day 4 and day 8 were fixed in 3.7% paraformaldehyde (Merck), rinsed in PBS (3 x for 5 min) and stored in PBS at 4°C until use. Whole-mount stainings were performed to visualize the actin cytoskeleton and the nuclei of PMA stimulated THP-1 cells cultured up to day 8 on scaffolds with different microarchitectures. Formaldehyde-fixed samples were washed in PBS and permeabilized in 0.5% Triton X-100 in PBS (Merck Serono) on a shaker. For F-actin staining, samples were incubated with 1:200 Phalloidin Atto-488 (Molecular Probes) in PBS overnight at 4°C. Scaffolds were rinsed in PBS and stained with 4′,6-diamidino-2-phenylindole (DAPI, Sigma Aldrich) for 5 min. After washing steps, the stained constructs were directly visualized with a confocal laser scanning microscope (Zeiss LSM 510 META, Zeiss). To determine overall cellular infiltration, scaffolds were embedded in optimal cutting temperature compound (Tissue-Tek^®^), cut in cross-sections (10 μm cryosections) and mounted on Polysine-coated glass slides (Thermo Fisher). Slides were washed in Milli-Q, stained with Weigert's iron hematoxylin (5 min, Sigma Aldrich), washed in running tap water (5 min) and visualized with bright-field microscopy. The average percentage of cellular infiltration was determined by 6 individual measurements per representative image (*N* = 3 images/scaffold type) using Image J software (U.S. National Institutes of Health, Bethesda, MD, USA). The average infiltration depths were used to calculate the average fiber surface areas that were effectively exposed to the cells.

### Statistical Analysis

MDA and enzymatic activity data are presented as Tukey boxplots, visualizing the median, the distribution of the data and statistical outliers (indicated as dots). DNA, ELISA, and qPCR data are expressed as means ± standard deviation. qPCR data were logarithmically transformed before statistical analyses. One-way ANOVA with Bonferroni *post-hoc* test or a Kruskal-Wallis test with a Dunn's multiple comparison test were performed for normally distributed or not normally distributed data, respectively. Statistical analysis was performed with Prism software (GraphPad, La Jolla, CA, USA), and differences were considered significant for *p* < 0.05. ^*^, ^**^, ^***^ denote significant differences for *p* < 0.05, 0.01, and 0.001, respectively.

## Results

### PCL-BU Scaffolds With Distinct Microarchitectures

**Figure 2:**
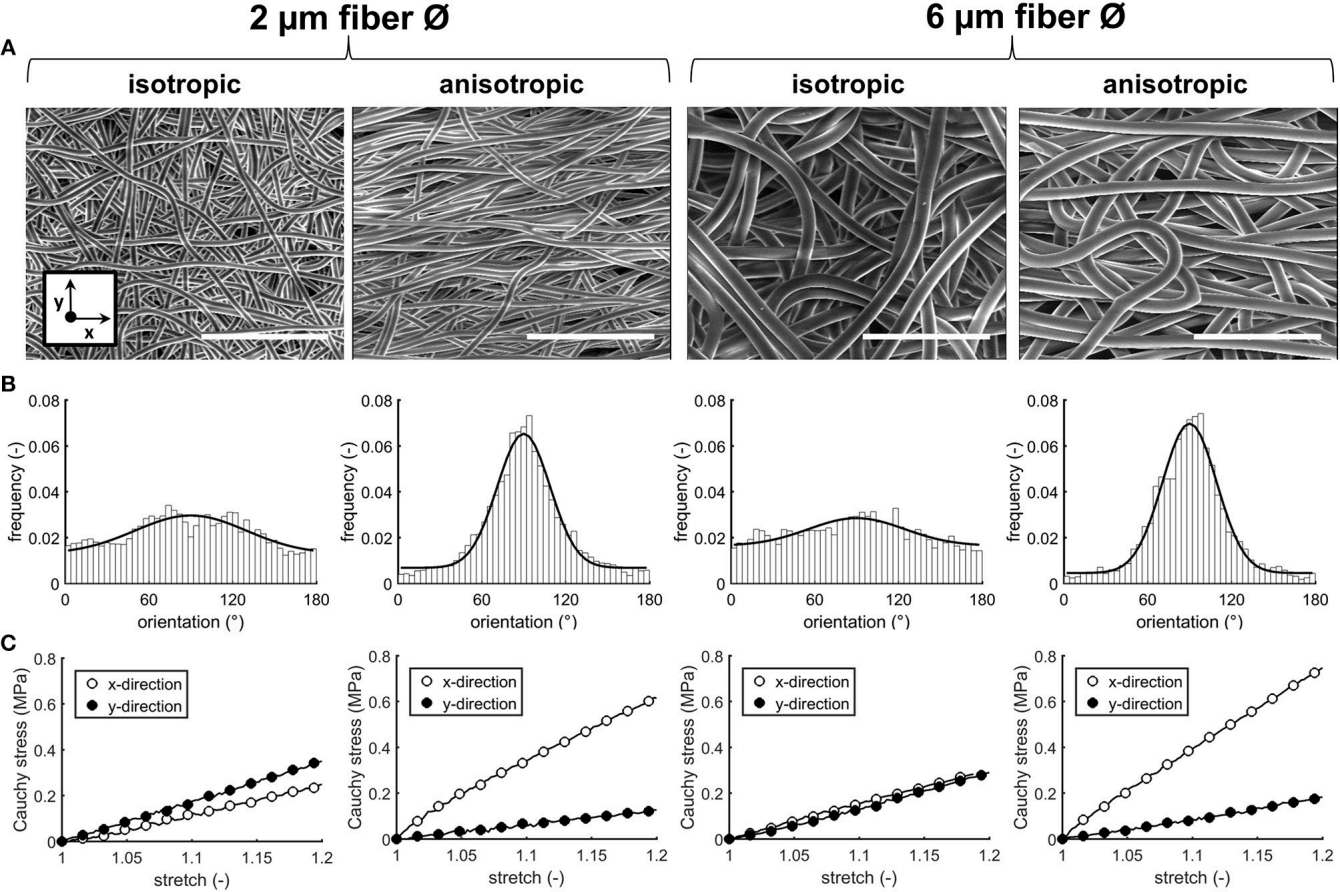
Scaffold characteristics. SEM images of the electrospun PCL-BU scaffolds with different fiber diameter and alignment. Scale bars, 50 μm **(A)**. Frequency histograms with fitted Gaussian distributions to visualize the degree of fiber alignment (90° = main axis) **(B)**. Average cauchy stress-stretch curves of the electrospun microarchitectures (*n* = 2/group, x = circumferential direction of the electrospinning mandrel; y = axial direction of the electrospinning mandrel) **(C)**.

Four types of electrospun scaffolds with comparable thickness, but different fiber diameter (average fiber Ø of ~2 or 6 μm), alignment (isotropic vs. anisotropic) and surface-to-volume ratios were created (Table 4, Figure 2A). Quantification of fiber orientation confirmed either an isotropic or anisotropic fiber orientation. Particularly, for the anisotropic scaffolds, the majority of the fibers were oriented in the circumferential direction of the electrospinning mandrel, while the isotropic scaffolds exhibited almost equal distributions in all directions (Figure 2B). The 6 μm fiber Ø scaffolds contained larger pore sizes in comparison to the 2 μm fiber Ø scaffolds (Table 4). Moreover, as a result of fiber directionality, the anisotropic scaffolds exhibited more elongated pore geometries as indicated by the larger width-to-height ratios (Table 4).

**Table 4:** Fiber alignment, diameter, pore size and width-height ratio, thickness, and surface to volume ratio of the electrospun scaffolds.

**Alignment**	**Fiber Ø [μm]**	**Pore size [μm^**2**^]**	**Width-height ratio pores**	**Thickness [μm]**	**Surface-to-volume ratio [m^**2**^/cm^**3**^]**
Isotropic	2.1 ± 0.3	2.6 ± 1.3	2.1 ± 0.8	167 ± 11	1.90
	5.7 ± 0.3	11.0 ± 6.2	5.0 ± 1.9	176 ± 16	0.70
Anisotropic	2.2 ± 0.2	2.7 ± 1.5	2.1 ± 0.9	123 ± 7	1.82
	5.7 ± 0.5	7.3 ± 3.4	5.7 ± 3.7	134 ± 7	0.70

Biaxial tensile testing revealed that scaffolds with anisotropic fibers displayed an anisotropic mechanical behavior with a higher stiffness in the direction circumferential to the electrospinning mandrel compared to the axial direction, as shown by the Cauchy stress-stretch curves (Figure 2C).

### Microarchitecture Induces Minor Variance in Macrophage Morphology

**Figure 3:**
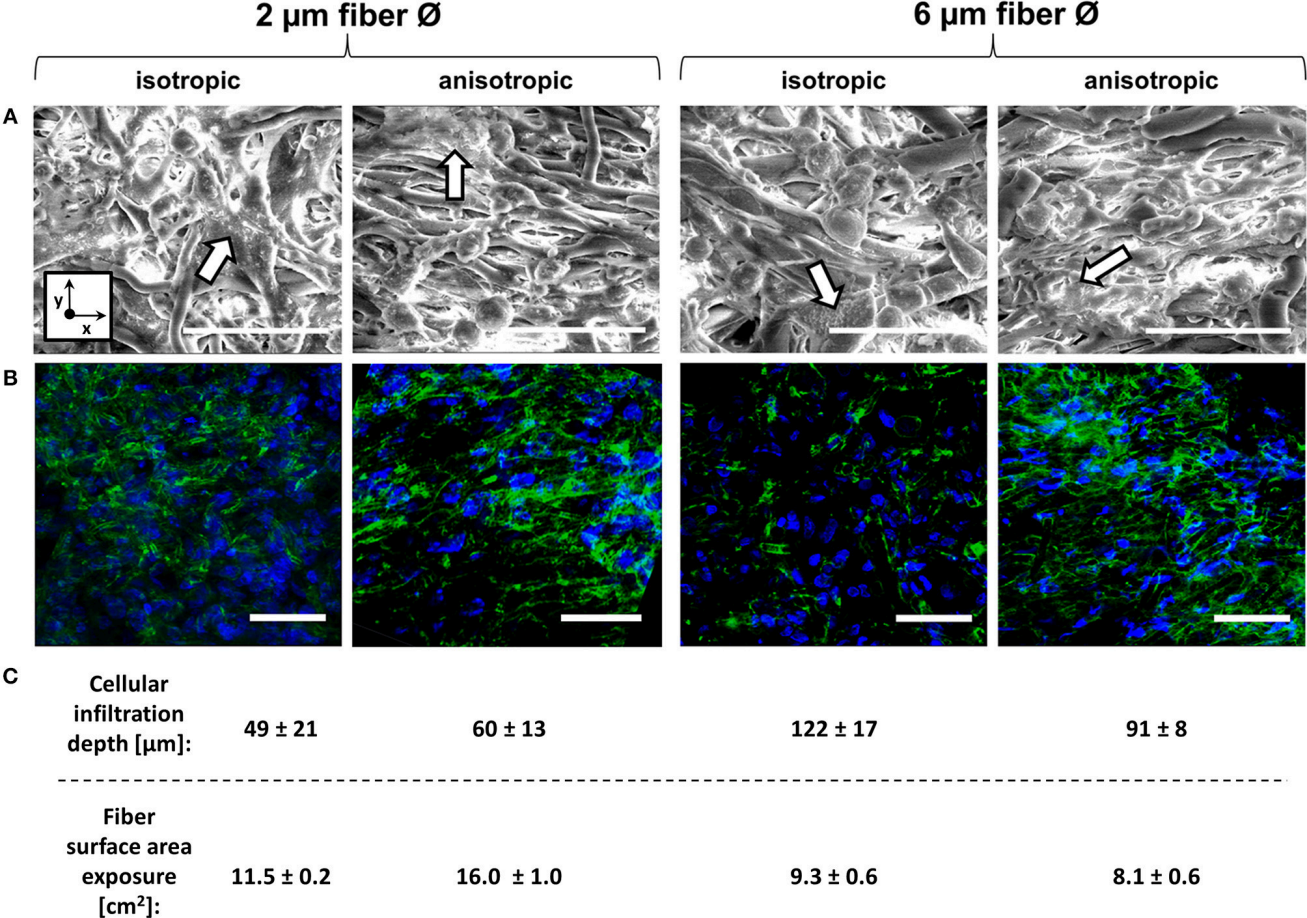
Macrophage morphology on different scaffold microarchitectures. Representative SEM images of macrophages cultured for 8 days on the different scaffold microarchitectures. Arrows identify areas of cell clustering. (x = circumferential direction of the electrospinning mandrel; y = axial direction of the electrospinning mandrel) Scale bars, 30 μm **(A)**. Representative confocal images showing the actin cytoskeleton of macrophages cultured up to 8 days on the different scaffold microarchitectures (actin in green; cell nuclei in blue). Scale bars, 50 μm **(B)**. The average cellular infiltration depth (μm) and calculated fiber surface exposure (cm^2^) per scaffold type **(C)**.

Cell seeding resulted in homogeneous cell distribution throughout the top layer of the scaffold for all scaffold types. After 4 days of culture, macrophages predominantly adopted a rounded shape on isotropic and anisotropic scaffolds, irrespective of the fiber diameter (data not shown). At day 8, cells tended to spread and cluster together, regardless of the fiber diameter and orientation (Figure 3A). Visualization of the actin cytoskeleton revealed cellular alignment in the anisotropic oriented 2 μm Ø scaffolds, and, to a lower extent, in the 6 μm Ø anisotropic scaffolds. All isotropic scaffolds, regardless of the fiber diameter, exhibited randomly oriented cells (Figure 3B). Cells infiltrated deeper into the 6 μm fiber Ø scaffolds (122 ± 17 μm isotropic vs. 91 ± 8 μm anisotropic) in comparison to the 2 μm fiber Ø scaffolds (49 ± 21 μm isotropic vs. 60 ± 13 μm anisotropic) regardless of the fiber alignment (Figure 3C). Consequently, the fiber surface area which was effectively exposed to cells was estimated to be 11.5 ± 0.2 cm^2^ and 16.0 ± 1.0 cm^2^ for the 2 μm fiber Ø isotropic and anisotropic scaffolds, respectively, and 9.3 ± 0.6 cm^2^ and 8.1 ± 0.6 cm^2^ for the 6 μm fiber Ø isotropic and anisotropic scaffolds, respectively.

DNA quantification revealed higher cellular presence for the 6 μm Ø isotropic scaffolds when compared to the other scaffold architectures at day 4, while no significant differences were detected between the various microarchitectures at day 8 (see Supplementary Figure S2, top). In presence of biochemical stimuli, the DNA content of LPS/IFN-γ stimulated cells was significantly lower than the DNA content measured for all the other groups in 2D (see Supplementary Figure S2, bottom). In 3D, comparable DNA amounts were detected for all the groups at day 4, while significantly less DNA content was measured in the LPS/IFN-γ stimulated cells at day 8.

### Scaffold Microarchitecture Affects Macrophage-Driven Degradation

**Figure 4:**
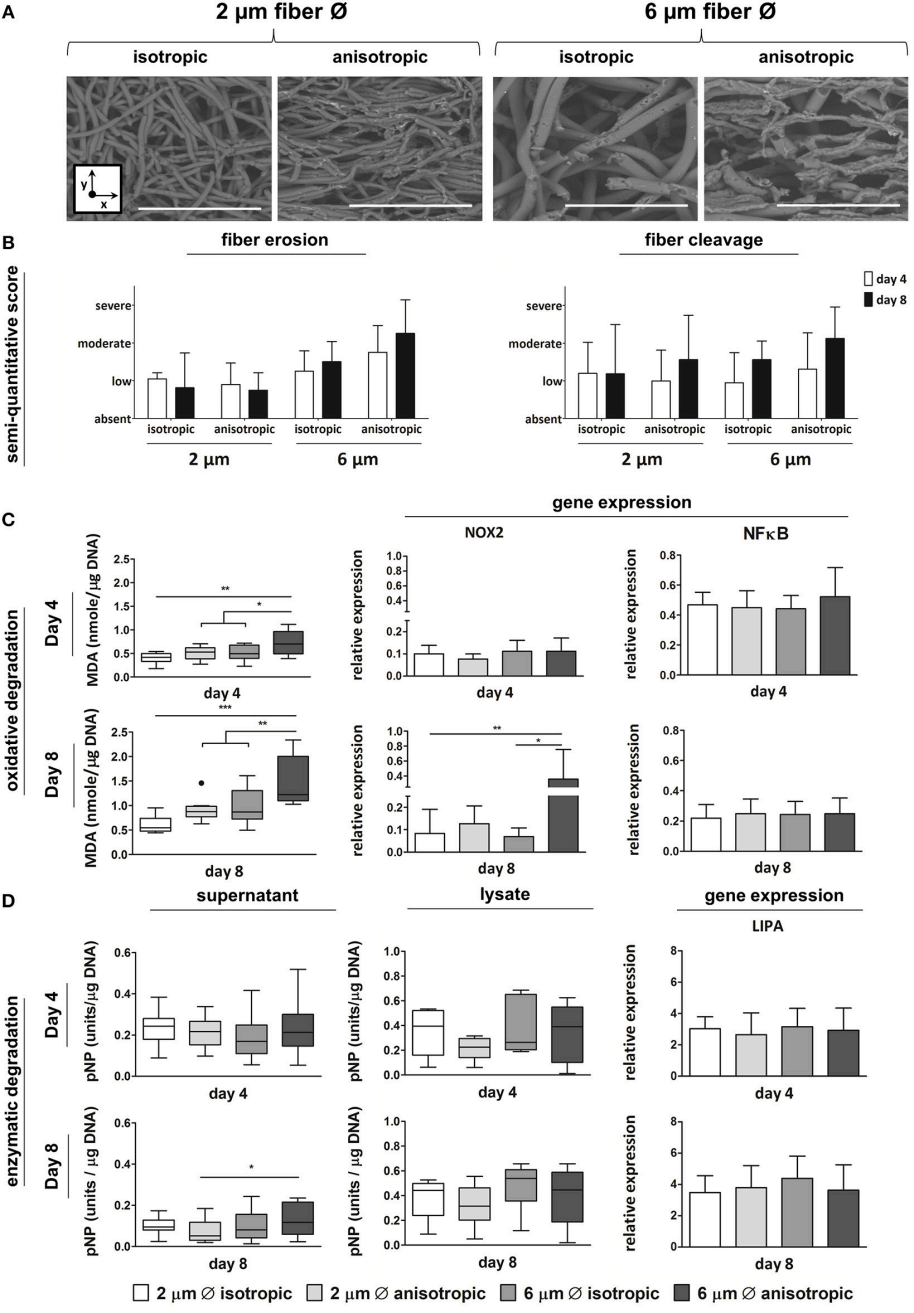
Macrophage-driven biomaterial degradation. Representative SEM images of decellularized scaffolds at day 8. Scale bars, 40 μm; x = circumferential direction of the electrospinning mandrel; y = axial direction of the electrospinning mandrel **(A)**. Semi-quantitative scoring of fiber erosion and cleavage seen in each scaffold type in time **(B)**. Oxidative degradation of the different scaffold microarchitectures in terms of malondialdehyde (MDA) presence and expression of the oxidative genes NOX-2 and NFkB (compared to CYC-1) at day 4 (top row) and day 8 (bottom row) **(C)**. Enzymatic degradation of the synthetic scaffolds investigated via esterase assays on both cell supernatant and lysate (1 unit = 1 nmol pNP released from pNPB per minute), and gene expression (compared to CYC-1) of LIPA at both day 4 (top row) and day 8 (bottom row) **(D)**. A dot represents a statistical outlier. *p < 0.05; **p < 0.01; ***p < 0.001, *N* ≥ 6/group/time point.

Anisotropic scaffolds with 6 μm fiber Ø showed the most pronounced fiber erosion and cleavage, at both day 4 and day 8, as determined by SEM analysis and scoring (Figures 4A,B). Increased levels of lipid peroxidation, as indicated by increased MDA levels, were observed in scaffolds with 6 μm, aligned fibers, both at day 4 and day 8 (Figure 4C). At the gene level, no significant differences were seen in the expression of NOX-2 at day 4, while significant upregulation was detected for cells cultured up to day 8 on the 6 μm fiber Ø anisotropic scaffolds only. Expression levels of the NFκB gene were comparable for all scaffold architectures, at both day 4 and day 8 (Figure 4C). Enzymatic activity, which was measured both in the supernatant and in the cell lysate, was significantly higher in the supernatant of the 6 μm fiber Ø anisotropic scaffolds, when compared to the 2 μm fiber Ø anisotropic scaffolds. However, overall enzymatic activity in the study's timespan of 8 days was low in all groups without any other significant differences between microarchitectures (Figure 4D). No significant differences in LIPA gene expression were observed between the experimental groups, at both day 4 and day 8 (Figure 4D).

### Differences in Degradation Cannot Be Explained by Canonical M1 or M2 Macrophage Polarization

#### Gene Expression

**Figure 5:**
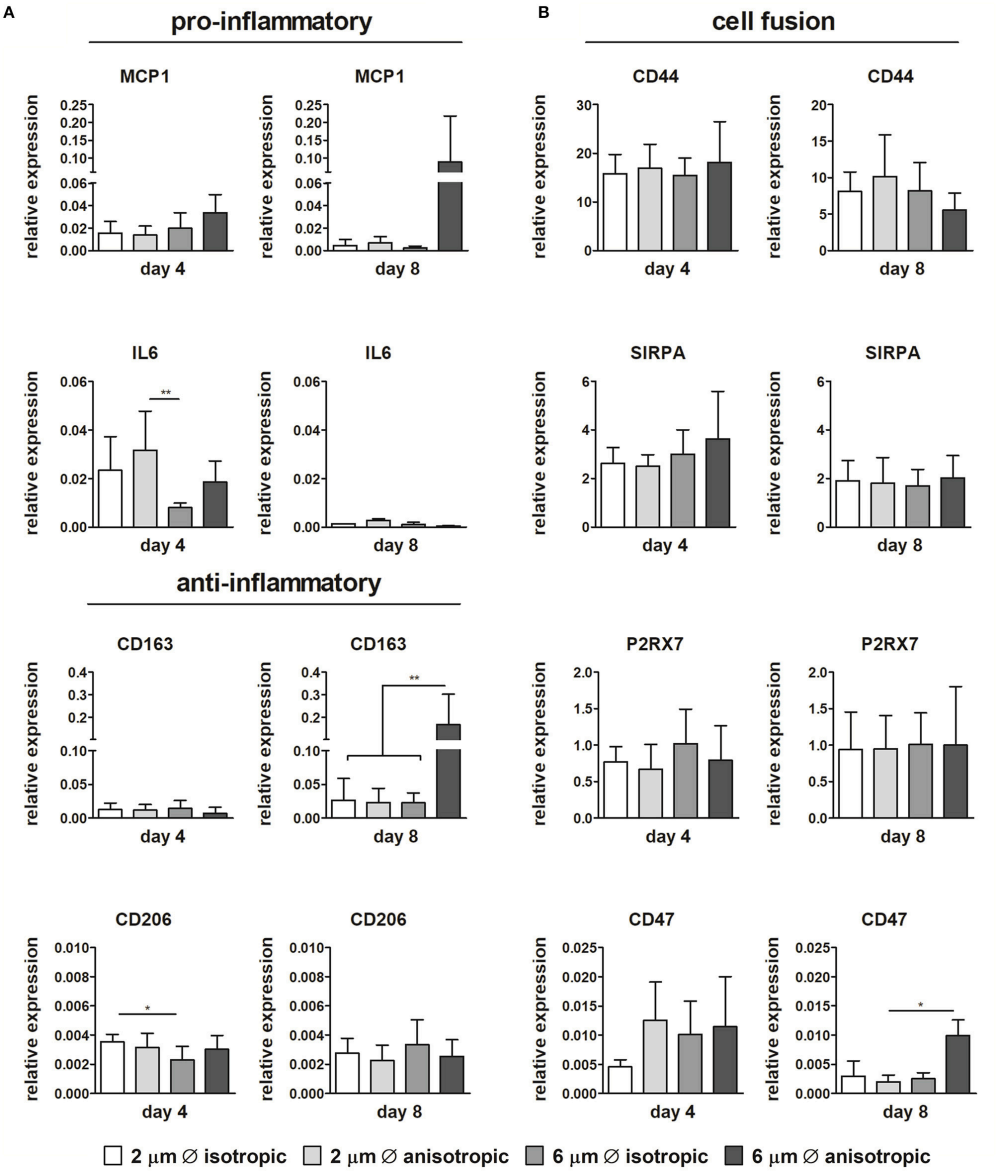
Gene expression profiles. Relative expression of pro- and anti-inflammatory genes in macrophages cultured up to day 4 and day 8 on scaffolds with different microarchitectures **(A)**. Relative gene expression of cell fusion markers CD44, SIRPA, the P2x7 receptor, and CD47 **(B)**. CYC-1 was selected as the reference gene. *p < 0.05; **p < 0.01. *N* ≥ 3/group/time point.

Limited differences in gene expression were observed for the pro-inflammatory and anti-inflammatory markers depending on the scaffold microarchitecture (Figure 5A). The expression of IL6 significantly increased in scaffolds with 2 μm Ø anisotropic fibers in comparison to the 6 μm isotropic scaffolds at day 4. However, at day 8, this trend has disappeared. While the relative expression of pro-inflammatory MCP1 and anti-inflammatory CD163 revealed no differences between the scaffold microarchitectures at day 4, marked upregulation was observed at day 8 for cells cultured on scaffolds with 6 μm Ø anisotropic fibers. For the anti-inflammatory CD206, a low but significantly higher expression was observed for the 2 μm Ø isotropic scaffolds in comparison to the 6 μm Ø isotropic scaffolds at day 4, while no significant differences were seen between the groups at day 8 (Figure 5A).

No considerable differences in expression could be detected between the different microarchitectures for CD68, CCR7, TNFα, IL10, TGFB1, MMP9 (see Supplementary Figure S3). With respect to cell fusion, no significant differences in relative expression of CD44, SIRPA, and P2X7 were observed amongst the scaffold microarchitectures. Instead, CD47 gene expression was upregulated for the 6 μm Ø anisotropic group in comparison to the other groups at day 8, although overall expression was low (Figure 5B).

#### Protein Secretion

**Figure 6:**
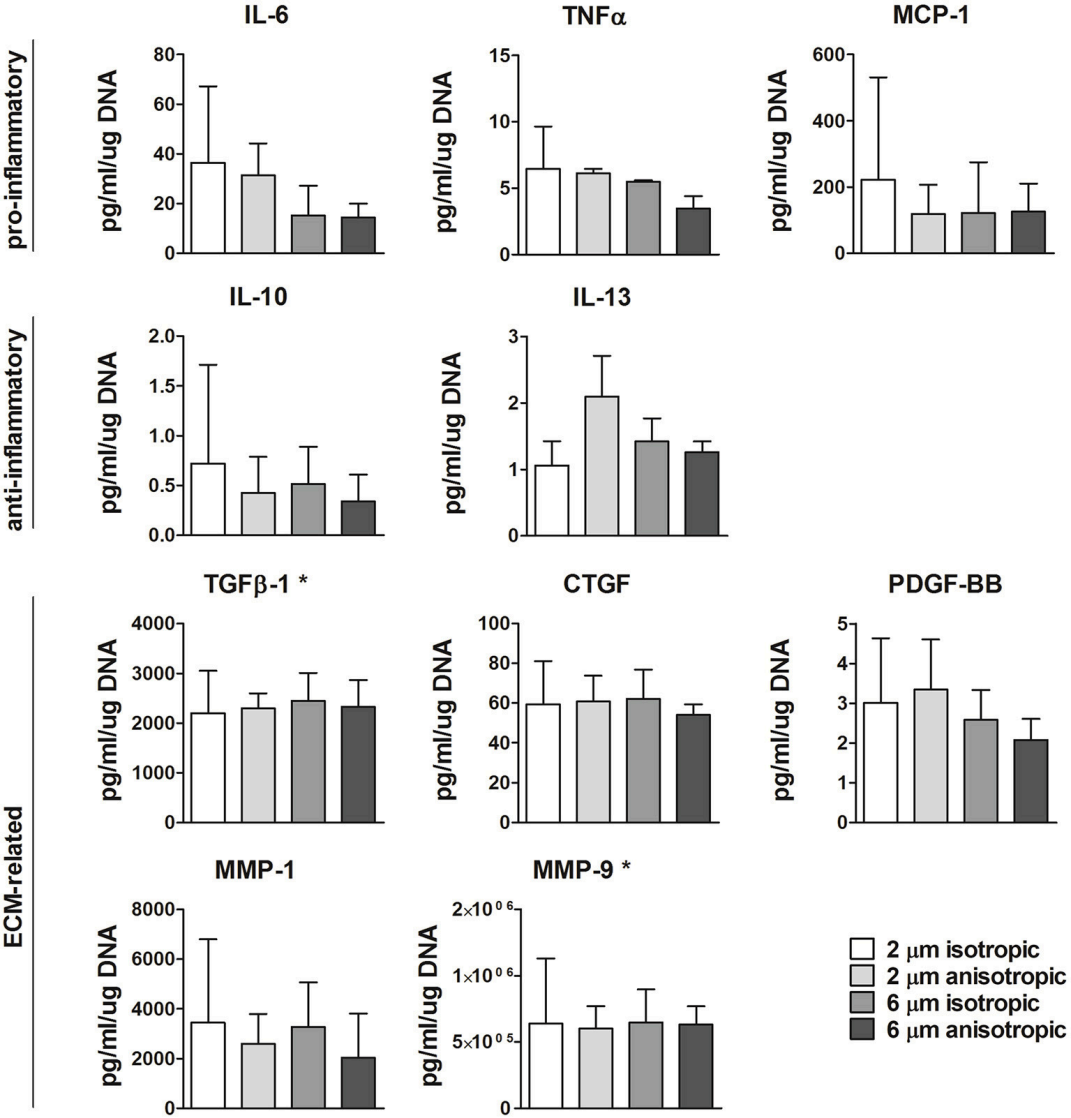
Protein secretion. Secretion levels of pro- or anti-inflammtory, and ECM-related proteins for macrophages cultured up to day 8 on scaffolds with different microarchitectures. *Values were extrapolated beyond the standard range. *N* ≥ 3/group/time point.

Protein secretion was quantified in the supernatant at day 8 from the cells cultured on the different scaffold microarchitectures. As shown in Figure 6, moderate yet not significant downregulation of IL-6 was observed with increasing fiber diameter. The anti-inflammatory IL-13 was mostly produced by cells cultured on 2 μm Ø anisotropic scaffolds, with no significant differences between the other groups. No significant differences were observed between the scaffold microarchitectures for all the other pro- or anti-inflammatory proteins evaluated, i.e., TNF-α, MCP-1, IL-10. Secretion levels of the ECM related proteins TGFβ, CTGF, PDGF-BB, MMP-1, and MMP-9 were comparable for all the scaffold microarchitectures (see Figure 6). Secretion levels of IFN-γ, FGFb, and elastase were below the limit of detection (data not shown).

### Cytokine Addition Results in Distinct Macrophage Phenotypes in 2D and in 3D

**Figure 7:**
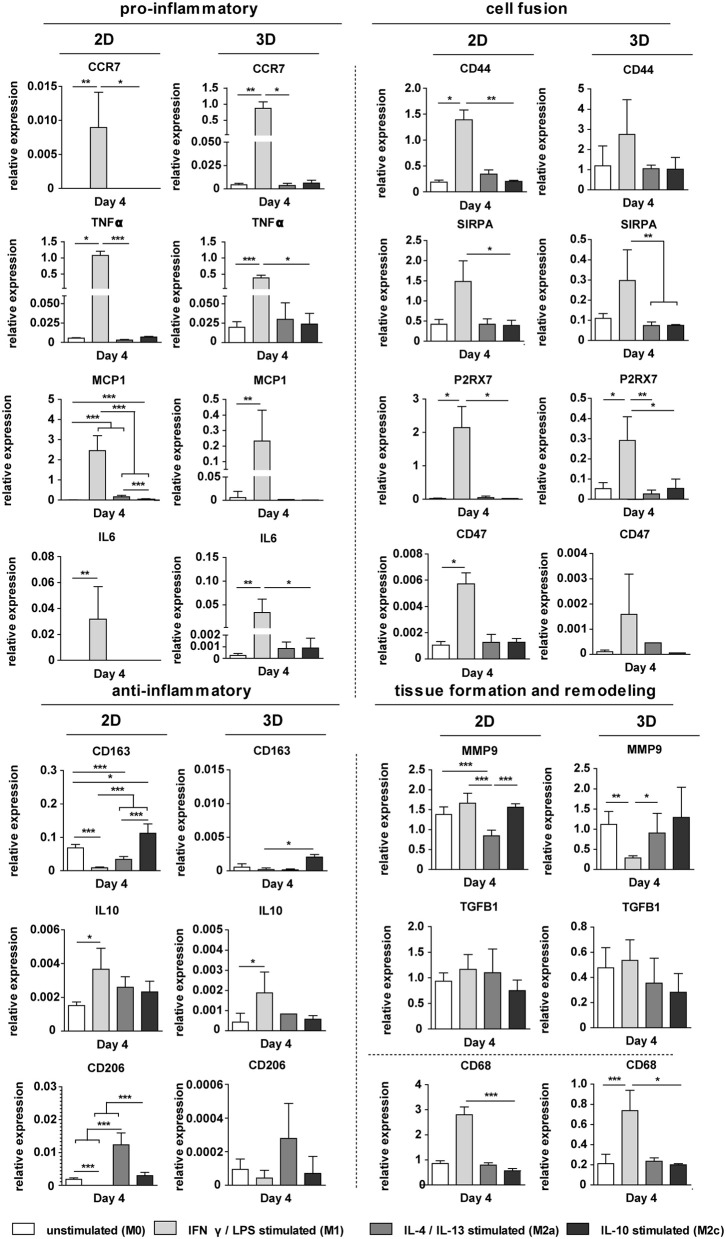
Gene expression profiles in 2D and 3D. Relative expression of pro- and anti-inflammatory genes, ECM-related genes, and genes involved in cell fusion for biochemically stimulated macrophages cultured on polysterene (2D) or on 6 μm fiber Ø isotropic scaffolds (3D) for 4 days. GAPDH was selected as the reference gene. *p < 0.05; **p < 0.01; ***p < 0.001, *N* ≥ 6/group/time point.

The exogenous addition of biochemical cues resulted in distinct macrophage phenotypes, both in 2D and in 3D scaffolds, as revealed by the gene expression profiles [see Supplementary Figure S4 (3D, day 8) and Figure 7 (2D and 3D, day 4), respectively]. Macrophages stimulation with the pro-inflammatory LPS/IFN-γ cytokines resulted in significant upregulation of the pan-macrophage CD68 gene, IL6 gene and the pro-inflammatory genes CCR7, TNFα, MCP1, both in 2D and 3D. Stimulation with the anti-inflammatory cytokines IL-4/IL-13 or IL-10 resulted in upregulation of CD206 or CD163, respectively, at day 4 and 8, in 2D and 3D. MMP9 was significantly downregulated after IL-4/IL-13 exposure at both day 4 (2D) and day 8 (3D). No significant differences were observed in TGFB1 expression between the groups at day 4. However, at day 8, TGFβ was significantly downregulated in case of IL-4/IL-13 or IL-10 stimulation. The cell fusion markers CD44, SIRPA, P2X7, and CD47 were significantly upregulated in the LPS/IFN-γ stimulated group (3D) at both day 4 and 8.

### Biochemically Polarized Macrophages Display Different Degradative Profiles

**Figure 8:**
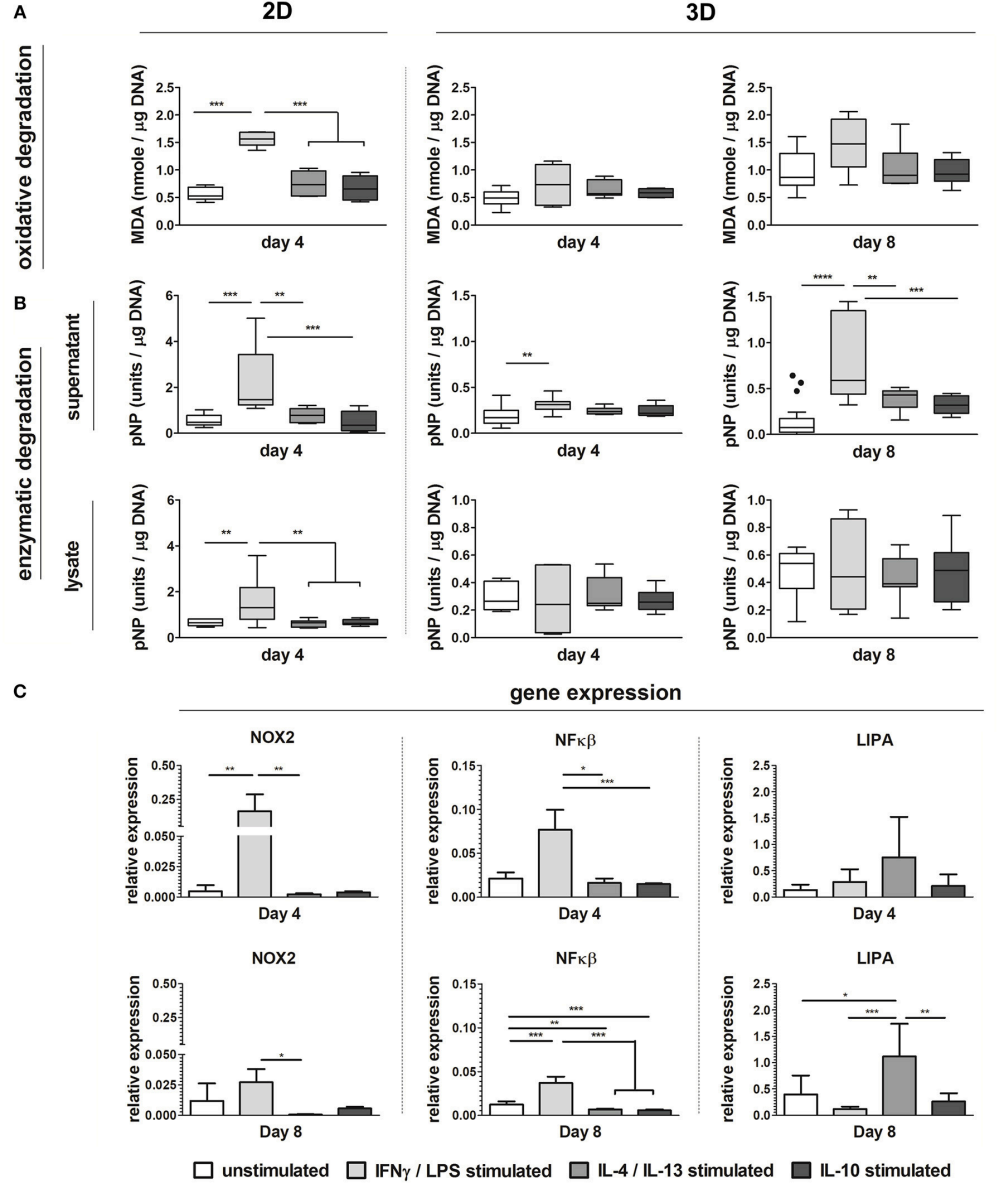
Macrophage phenotype-driven biomaterial degradation. Boxplots visualizing MDA production (oxidative stress) of unpolarized macrophages (M0), LPS/IFN-γ stimulated macrophages (M1), IL-4/IL-13 stimulated macrophages (M2a), and IL-10 stimulated macrophages (M2c) cultured up to 4 days on tissue culture treated well plates (“2D”) or on scaffolds with 6 μm Ø isotropic fibers (“3D”) for 4 days and 8 days **(A)**. Enzymatic activity detected in the supernatant and cell lysate of M0, M1, M2a, and M2c macrophages cultured up to day 4 (2D and 3D) and day 8 (3D) (1 unit = 1 nmol pNP released from pNPB per minute) **(B)**. Gene expression (compared to GAPDH) of NOX2, NFκB, and LIPA for M0, M1, M2a, and M2c macrophages cultured on scaffolds with 6 μm Ø isotropic fibers **(C)**. MDA and enzyme activity values are corrected for DNA content. *p < 0.05; **p < 0.01; ***p < 0.001. *N* ≥ 4/group/time point.

Biochemically polarized macrophages displayed differences in degradative capacity, in terms of enzyme activity as well as oxidative stress, in both 2D and 3D environments (6 μm Ø, isotropic scaffolds) (Figure 8). Particularly, in 2D at day 4, LPS/IFN-γ stimulated macrophages gave the highest levels of MDA and enzymatic activity, in both supernatant and cell lysate, when compared to the other groups (Figures 8A,B). Similarly, in 3D and especially at day 8, MDA was abundantly expressed by LPS/IFN-γ stimulated macrophages (M1) when compared to all the other groups. However, differences with the other groups did not reach statistically significant differences (Figure 8A). Enzymatic activity in the supernatant of LPS/IFN-γ stimulated macrophages in 3D was significantly higher compared to unpolarised macrophages (M0), at both day 4 and day 8. At day 8, the enzymatic activity in this group was also significantly higher than the IL-4/IL-13 stimulated macrophages (M2a) and the IL-10 stimulated macrophages (M2c). No significant differences were seen in the enzymatic activity of the cell lysates between the groups (Figure 8B).

At the gene level, significant upregulation of NOX2 and NFκB genes was detected for LPS/IFN-γ stimulated macrophages (M1) in 3D, at both day 4 and 8, compared to the other groups. LIPA was moderately and significantly upregulated by macrophage stimulation with IL-4/IL-13 at day 4 and day 8, respectively (Figure 8C).

## Discussion

In this study, we performed a detailed analysis of the effect of scaffold microarchitecture (fiber diameter and alignment) on macrophage-driven scaffold degradation. PCL-BU elastomeric scaffolds with two different fiber diameters, 2 and 6 μm, and alignments, isotropic and anisotropic, were seeded with human macrophages and cultured up to 8 days *in vitro*. First and foremost, it was demonstrated that the scaffold microarchitecture has a significant effect on macrophage-driven degradation. Specifically, 6 μm fiber Ø anisotropic scaffolds showed the most pronounced oxidative degradation, as revealed by the marked upregulation of the NOX2 gene, increased levels of lipid peroxidation, and the pronounced fiber erosion and cleavage detected via SEM analysis. With respect to enzymatic degradation, negligible differences were observed between the various scaffold microarchitectures over time. Surprisingly, while biochemically polarized macrophages displayed a degradative profile dependent on their polarization state (both in 2D and in 3D), the observed differences in degradation instigated by the scaffold microarchitecture could not be attributed to the canonical M1-M2 polarization spectrum. This suggests that the scaffold microarchitecture uniquely affects macrophage-driven degradation.

Various studies have demonstrated a dependency of the degradative capacity of macrophages, and oxidative degradation particularly, on biochemically induced macrophage phenotype. For example, it was shown that LPS-treated pro-inflammatory macrophages are capable of exerting oxidative stress, releasing NO and ROS (Groemping et al., 2003; Tan et al., 2016). Moreover, in PMA-activated macrophages, high levels of ROS were associated with high expression of pro-inflammatory cytokines (Song et al., 2015). In contrast to M1 macrophages, M2 activation was hypothesized to be accompanied by reduced NAPDH oxidase (NOX2) activity, ROS and NO generation (Tan et al., 2016). In line with these observations, our study demonstrated that LPS/IFN-γ treatment of macrophages in 2D and 3D led to a pro-inflammatory gene expression profile and induced a significant upregulation of lipid peroxidation, overexpression of the oxidative genes NOX2 and NFκB, and increased enzymatic activity, when compared to the unpolarized (M0), alternatively-activated (M2a) or reparative macrophages (M2c). Indirect confirmation of the M1-induced ROS production was found in the drastic decrease in viability observed for the LPS/IFN-γ treated macrophages in 2D (at day 4) and in 3D (at day 8). In fact, in previous studies it was shown that although macrophages develop ROS-resistance in fighting against overwhelmed intracellular ROS level, ROS-induced self-destruction mechanisms might still not be completely prevented (Essler et al., 2009; Bauer et al., 2011; Dey et al., 2014). Correspondingly, the unexpected upregulation of IL10 in the M1 group can be related to a cell-defense mechanism to suppress intra-cellular NO production and maintain cell metabolic equilibrium (Baseler et al., 2016).

Besides exogenous addition of cytokines, the scaffold microarchitecture can profoundly affect macrophage polarization, e.g., due to changes in cell shape, podosome configurations, and cytoskeletal reorganizations (Li et al., 2011; Versaevel et al., 2012; Ghrebi et al., 2013; Cabanel et al., 2015; Chen et al., 2016; Das Gupta et al., 2016). To illustrate, larger fiber Ø and pore sizes in the μm range, together with microarchitecture-induced cell elongation, were proven to contribute to enhanced alternative macrophage polarization (M2) and downstream tissue regeneration (Sanders et al., 2002; Saino et al., 2011; Garg et al., 2013; McWhorter et al., 2013; Wang et al., 2014). Thus, we hypothesized that scaffold microarchitectures would influence macrophage polarization and, consequently, scaffold degradation. More specifically, selective polarization of macrophages into a pro-inflammatory phenotype was expected in scaffolds with the smaller isotropic fiber diameter (Ø 2 μm). However, gene expression and protein analysis revealed limited differences in macrophage phenotypes for the different scaffold microarchitectures, which suggests that: (i) the chosen microarchitectures did not induce selective M1 or M2 polarization, and that (ii) the observed differences in the degradation kinetics of scaffolds are related to other mechanisms rather than the polarization of macrophages into the canonical M1-M2 phenotypes.

The lack of microarchitecture dependent M1 or M2 polarization (i) might be explained by the choice of 2 and 6 μm as “small” and “large” fiber diameters, respectively. Previously, Wang et al. reported an increased presence of RAW264.7 derived M2 macrophages for PCL scaffolds with fiber diameters of 5.59 μm, when compared to scaffolds with 0.69 μm fiber diameter, both *in vitro* and *in vivo* (Wang et al., 2014). Similarly, Garg et al. demonstrated a positive correlation between increasing fiber diameter and pore size and expression of M2 markers when culturing mouse bone marrow-derived macrophages on polydioxanone (PDO) scaffolds with fiber diameter of either 0.35 and 2.8 μm *in vitro* (Garg et al., 2013). The seemingly contrasting lack of differences in macrophage polarization in our study may be explained by the notion that we compared fiber diameters in the micrometer range only. We specifically opted for relatively large fibers, and concomitantly large pore sizes, to ensure sufficent cell infiltration into the scaffolds, which is an essential element to *in situ* tissue engineering. Hence, although potentially interesting from a fundamental point-of-view, scaffolds with fibers in the nanometer range, and corresponding small pores, are beyond the scope of this work. Although we did not see clear differences in polarization for the two fiber diameters tested, we did detect an increase in MCP1 and CD163 gene expression for the anisotropic 6 μm fiber Ø scaffolds. As ROS has been recognized as a regulating factor of MCP-1 induced monocyte recruitment (Hackel et al., 2013) and CD163 is associated with phagocytosis (Lurier et al., 2017), these results support our finding that microarchitecture influences macrophage degradative potential. In addition to the fiber alignment and fiber diameter, the variations in pore geometry may have played a role influencing the cellular response (Garg et al., 2013; McWhorter et al., 2013; McWhorter et al., 2015).

With respect to other cellular mechanisms possibly involved in scaffold degradation (ii), it was recently shown that NADPH-oxidase generated ROS promotes cell fusion and, vice versa, that many of the factors involved in cell fusion catalyzes ROS generation (Quinn and Schepetkin, 2009). Interestingly, it was also reported by Anderson et al. that the adherent precursors of the FBGCs exhibited macrophage polarization profiles that were not classified as M1 or M2, but possessed an intermediate expression profile (Anderson et al., 2008). To investigate whether cell fusion occurred in our experimental groups, we investigated the expression of the fusion markers CD44, CD47, SIRPA, and P2X7 (Quinn and Schepetkin, 2009; McNally and Anderson, 2011). Despite the lack of differences in expression levels of CD44, SIRPA, and P2X7 for the different microarchitectures, moderate upregulation of CD47 was observed for macrophages cultured on the 6 μm fiber Ø anisotropic group, which might directly relate to the pronounced degradation observed for that particular scaffold microarchitecture. However, a more extensive analysis of the impact of microarchitecture on cell fusion is recommended.

Apart from cellular mechanisms, material processing may induce changes in material crystallization, which in turn could affect degradation. More specifically, various studies have demonstrated that an accelerated mandrel rotation speed might induce crystallization (Jose et al., 2007; Edwards et al., 2010). Our scaffolds are composed of PCL-BU material, a thermoplastic elastomer with a poly-ε-caprolactone soft block and a bis-urea hard block. Both these blocks can crystallize. Differential scanning calorimetry (DSC) measurements on electrospun scaffolds show that the soft block has two broad crystalline melt transitions (at ± 15 and ± 60°C), while the hard block has one broad transition (at ± 120°C). These transitions are seen for both the isotropic and the anisotropic scaffolds, albeit with minor differences in recorded melting enthalpies, indicating no to minor variations in crystallinity between the different microarchitectures as a result of the electrospinning process (see Supplementary Figure S5). Therefore, we reason that the most pronounced degradation in the 6 μm fiber Ø anisotropic scaffolds is most likely to be a cell-mediated, rather than a material-morphology driven process. Moreover, we verified that the increased material degradation in the 6 μm fiber Ø anisotropic group was not a result of a larger surface-to-volume ratio, nor a larger fiber surface area effectively exposed to cells in this group.

In parallel with scaffold degradation, the timely formation of functional tissue is of utmost importance for successful outcomes of the *in situ* tissue engineering approach (Wissing et al., 2017). Thus, as an additional read-out, the effect of macrophage product secretion (e.g., degrading products and cytokines) on tissue deposition was assessed via exposure of fibroblasts, i.e., human vena saphena cells (HVSCs), to the supernatant of macrophages cultured on the degrading scaffold microarchitectures for 24 h (see Supplementary Figure S6). Remarkably, for fibroblasts exposed to the supernatant of macrophages cultured of the highly degrading anisotropic 6 μm fiber Ø scaffolds, significant upregulation of the αSMA gene was observed, which might indicate a shift of fibroblasts into a more contractile and synthetic phenotype. Accordingly, for the same group, Col type III was significantly upregulated, in parallel to the downregulation of the fibroblast collagenase MMP1, indicating enhanced tissue deposition. In addition, specific macrophage phenotypes, induced by exogenous addition of cytokines, were shown to promote a different HVSCs response. Exposure to the supernatant of LPS/IFN-γ stimulated pro-inflammatory macrophages downregulated Collagen type I and Collagen type III, while stimulating the expression of MMP1 and MMP2 genes. These observations are consistent with literature (Song et al., 2000; Ploeger et al., 2013), and suggest that a pro-inflammatory macrophage phenotype might suppress tissue formation by fibroblasts, with potential risks of early failure of fast-degrading grafts.

This study has some inherent limitations. Human THP-1 cell line-derived macrophages were used as a cell source. Even though THP-1 cells have become one of the most widely used cell lines in this field (Qin, 2012; Chanput et al., 2014), it was derived from the blood of a patient with acute monocytic leukemia, which sparks strong debates on the accuracy of THP-1 cells in mimicking monocytes and macrophages behavior, especially when compared to primary cell sources. The use of primary cells over THP-1 cells also has the inherent advantage of accounting for donor-to-donor variability, which, in this particular case, might also influence the degradation fate of the implanted scaffolds as degrading product secretion might change with age, gender and co-morbidity (Labow et al., 2001b). Moreover, implant degradation involves the simultaneous action of multiple phagocytes and interrelated degradation pathways (Labow et al., 2002; McBane et al., 2005, 2007). Neutrophils and monocytes might be able to initially shape degradation via the release of hydrolytic enzymes (serine proteases) (Labow et al., 1995) and ROS, like hypochlorous acid (HOCl) (Thomas and Fishman, 1986). These compounds pretreat the biomaterial, making it more or less vulnerable for the propagation of macrophage-driven degradation (Labow et al., 2001a, 2002). Comparably, the PMA that was used as a stimulant for monocyte-to-macrophage differentiation might have altered surface chemistry via protein kinase C-stimulated NADPH oxidase upregulation and reactive oxidant production (e.g., HOCl; Thomas and Fishman, 1986; Jackson et al., 1995; Labow et al., 1999).

The results of this study were obtained by using supramolecular PCL-BU scaffolds. Although the results should be verified for other types of materials, the results of our current study do emphasize the importance of using 3D cell cultures, and specifically macrophages, for assessing scaffold degradation *in vitro*, in addition to cell-free accelerated *in vitro* degradation tests, as previously described (Brugmans et al., 2015). Although the latter will render relevant information on the susceptibility of materials to either enzymatic or oxidative degradation, such cell-free assays do not account for the influence of the scaffold microarchitecture on cell-driven degradation. The relevance of this is apparent from a recent study by Kluin et al. in which the *in vivo* degradation of an electrospun elastomer was observed to be highly heterogeneous and dependent on the amount and phenotype of infiltrating immune cells (Kluin et al., 2017).

## Conclusions

In this study we demonstrate that the scaffold microarchitectural design (e.g., choice of fiber diameter and alignment) uniquely affects early macrophage-driven scaffold degradation, in a mechanism which is independent of changes conform to the generally accepted M1-M2 paradigm. These results are highly instrumental for the design of synthetic materials and for the tailoring of their degradation kinetics for *in situ* tissue engineering purposes. Moreover, these findings advocate the use of 3D cell-based assays to adequately assess biomaterial degradation *in vitro*.

## Author Contributions

The CRediT Taxonomy are as follows: TW, MB, CB, and AS: conceptualization; TW, VB, EvH, HJ, and AS: formal analysis; TW, VB, EvH, MvD, and HJ: investigation; CB: funding acquisition; CB and AS: supervision; TW, VB, and AS: visualization; TW and VB: writing—original draft preparation; TW, VB, EvH, MvD, MB, HJ, CB, and AS: writing—review and editing. All authors have approved the final article.

### Conflict of Interest Statement

MB is employed by Xeltis B.V. and HJ is employed by SyMO-Chem B.V. The remaining authors declare that the research was conducted in the absence of any commercial or financial relationships that could be construed as a potential conflict of interest.
