# Supplementary material for: Macrophage-Driven Biomaterial Degradation Depends on Scaffold Microarchitecture
Source: Front Bioeng Biotechnol. 2019 Apr 26;7:87. doi: 10.3389/fbioe.2019.00087 (PMC6497794; doi:10.3389/fbioe.2019.00087)
Supplement: Supplementary file 1 [file Data_Sheet_1.pdf]

Supplementary Material to:

Macrophage-driven biomaterial degradation depends on scaffold microarchitecture

Authors:

Tamar B. Wissing[1,2], Valentina Bonito[1,2], Eline E. van Haaften [1,2], Marina van Doeselaar [1], Marieke M. C.P. Brugmans[3], Henk M. Janssen[4], Carlijn V.C. Bouten[1,2], Anthal I.P.M. Smits[1,2*]

Affiliations:

[1] Department of Biomedical Engineering, Eindhoven University of Technology, Eindhoven, The Netherlands

[2] Institute for Complex Molecular Systems (ICMS), Eindhoven University of Technology, Eindhoven, The Netherlands

[3] Xeltis B.V., Eindhoven, The Netherlands

[4] SyMO-Chem B.V., Eindhoven, The Netherlands

* Corresponding author: A.I.P.M.Smits@tue.nl, Tel: +31 40 247 4738

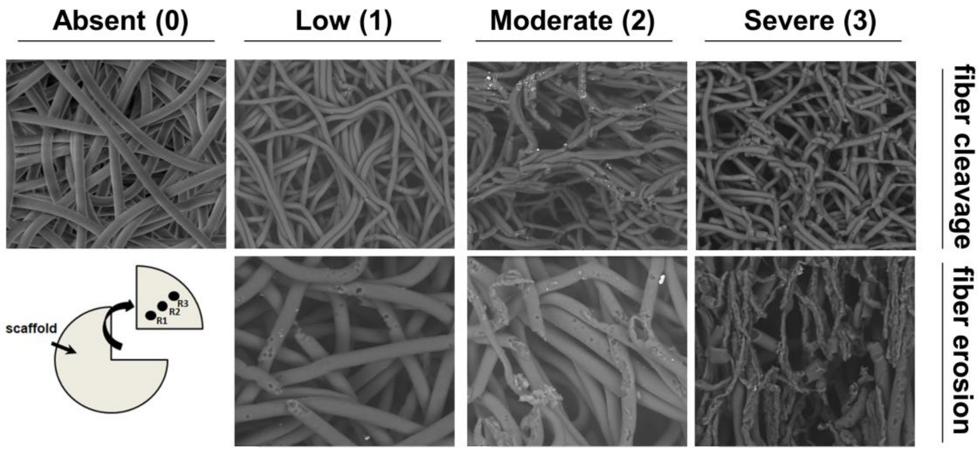

Supplementary Fig. S1. Reference images to semi-quantitatively assess fiber damage. The amount of fiber damage (e.g. erosion or cleavage) is subdivided into four categories: absent (0), low (1), moderate (2) and severe (3). Quarters of scaffolds (day 4 and 8) were decellularized and visualized with SEM on fixed locations (R1-3). Fiber damage was scored for each location by two researchers independently. N = 4-6 scaffolds / group / time point.

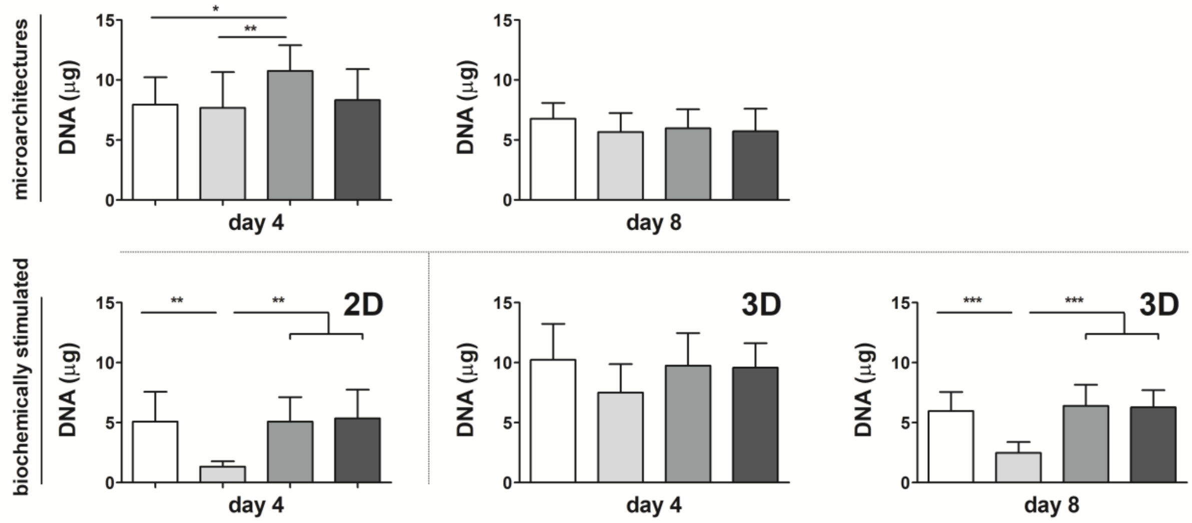

Supplementary Fig.S2. DNA quantification for macrophages cultured on different scaffold microarchitectures (top) and with different exogenous additions of cytokines (bottom, left in 2D, right in 3D using scaffolds with 6 µm fiber Ø isotropic fibers). * $p < 0.05$; ** $p < 0.01$; *** $p < 0.001$, $N \geq 6$ / group / time point.

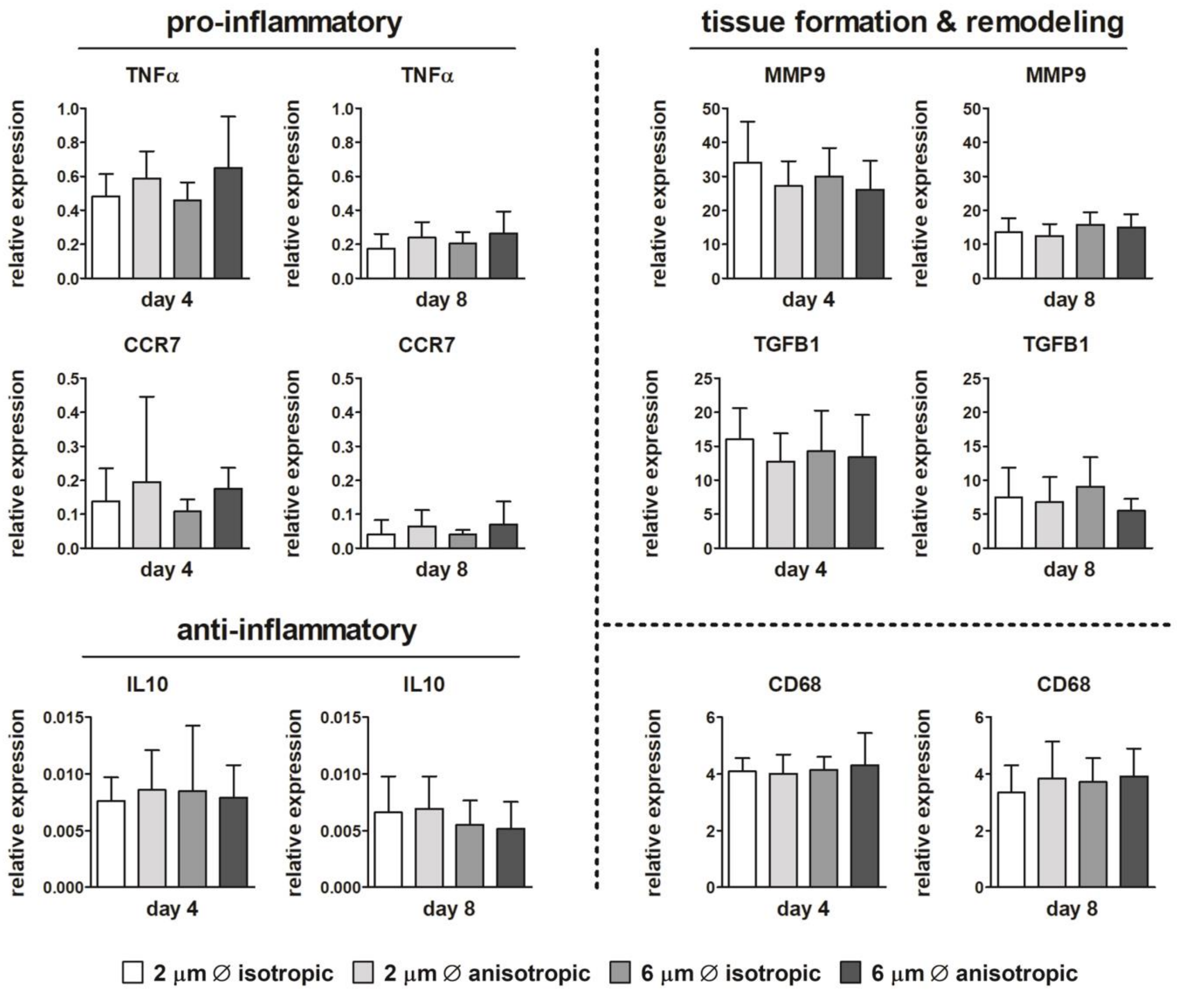

Supplementary Fig. S3. Gene expression profiles. A) Relative gene expressions (in comparison to CYC-1) of TNFα, CCR7, IL10, MMP9,TGFB1 and CD68 on day 4 and 8 for the macrophages seeded on the various microarchitectures. No differences in expression were detected for these markers in time. N ≥ 6 / group / time point. Data are presented as the mean ± SD.

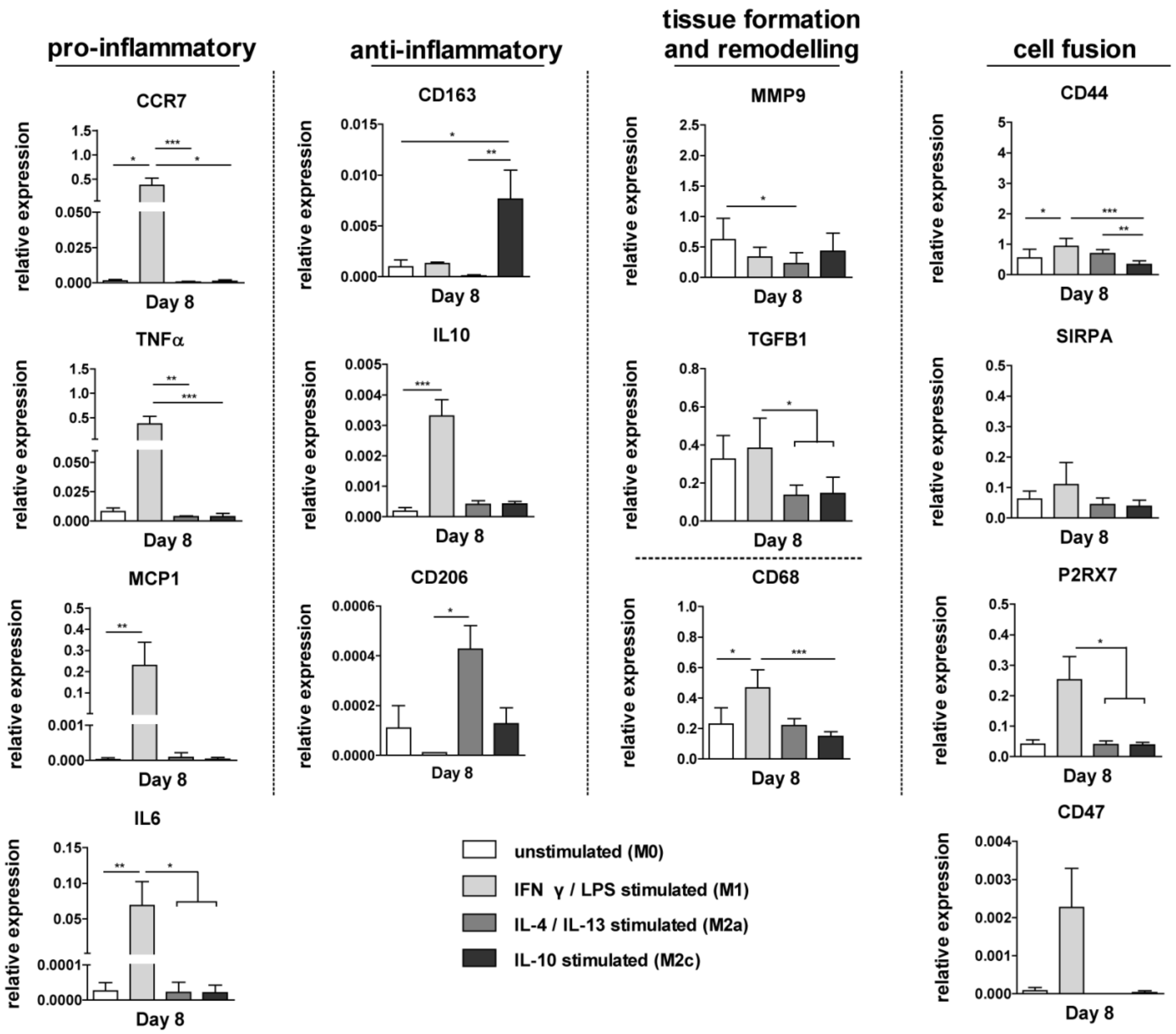

Supplementary Fig.S4. Gene expression profiles in 3D at day 8. Relative expression of pro- and anti-inflammatory genes, ECM-related genes, and genes involved in cell fusion for biochemically stimulated macrophages cultured on 6 µm fiber Ø isotropic scaffolds (3D) for 8 days. GAPDH was selected as the reference gene. * $p < 0.05$; ** $p < 0.01$; *** $p < 0.001$, $N \geq 6$ / group / time point.

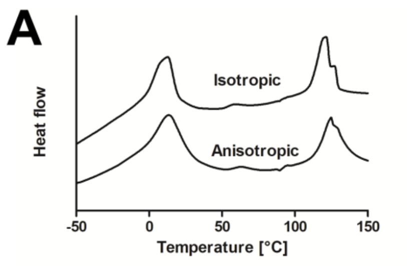

B

Alignment	Isotropic	Anisotropic
Enthaply [J/g]	8.6 ± 0.9	6.1 ± 0.6
Peak temperature [° C]	122.4 ± 1.8	124.7 ± 0.2

Supplementary Fig. S5. Differential scanning calorimetry (DSC) measurements. Representative DSC curves showing the crystalline melt transitions **(A)** and melting enthalpies and temperatures **(B)** of the bis-urea phase in isotropic and anisotropic electrospun PCL-BU scaffolds. N = 3 / group.

Differential scanning calorimetry (DSC) measurements were performed on a DSC Q2000 (TA instruments, USA). Prior to analysis, the electrospun scaffolds were weighed and thereafter sealed in Tzero aluminum pans. The scaffolds were cooled to -80°C and exposed to a heating/cooling cycle (-80°C to 160°C, 10°C/min), where after peak temperature (max temperature peak) and enthalpy (the surface under the peak) were determined using Universal Analysis software (V4.5A, TA Instruments).

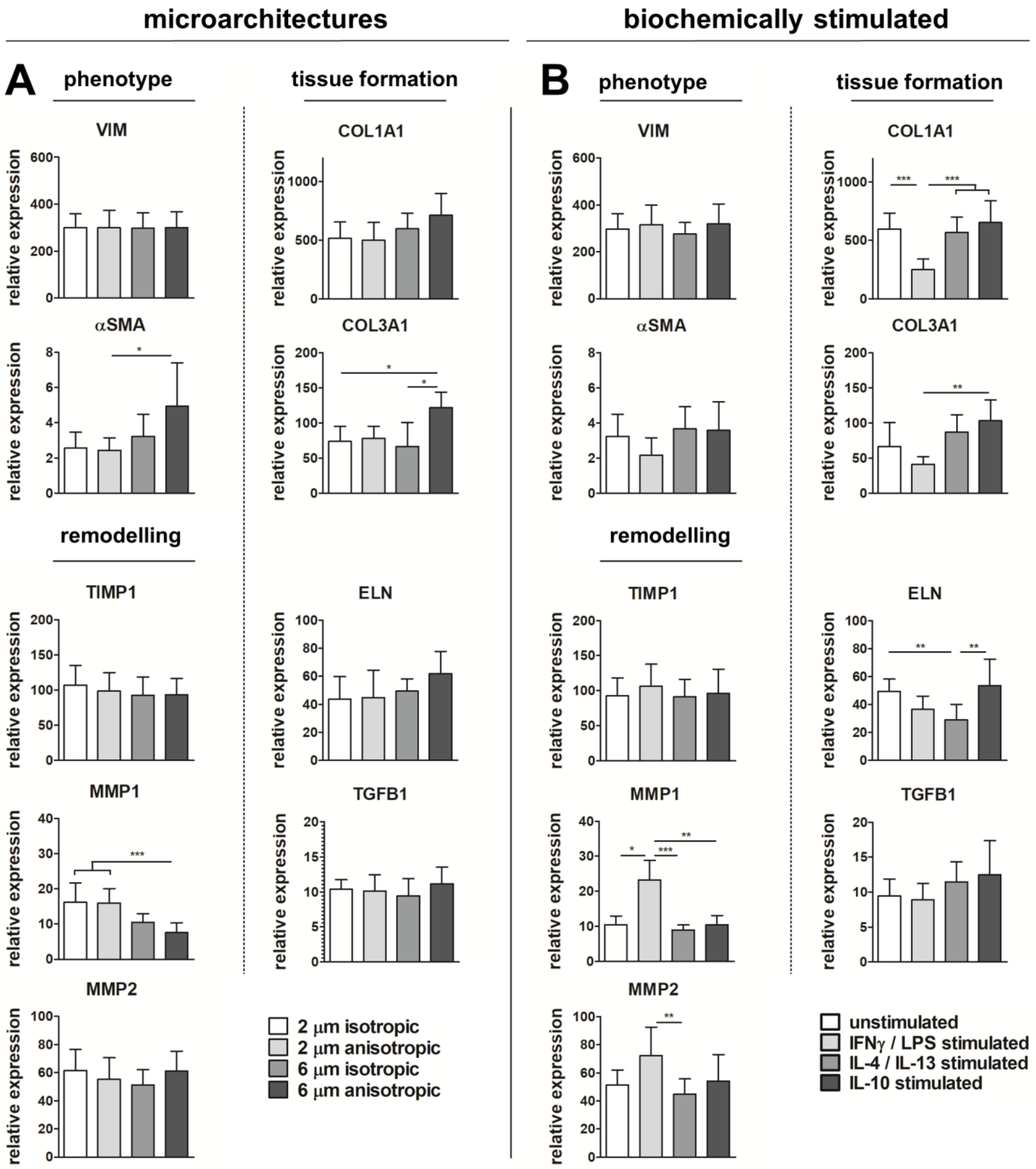

Supplementary Fig.S6. Gene expression profiles of HVSC exposed to macrophage-conditioned media of the experimental groups **(A)** or the biochemically stimulated control groups **(B).** Relative expression (compared to CYC-1) of phenotypical genes (VIM, αSMA), tissue-related genes (COL1A1, COL3A1, ELN, TGFB1), and genes involved in tissue remodeling (TIMP1, MMP1, MMP2) for HVSCs exposed to the supernatants for 24 hrs. GAPDH was selected as the reference gene. * $p < 0.05$; ** $p < 0.01$; *** $p < 0.001$. $N \geq 8$ / group.

Vascular derived human vena saphena cells (HVSCs) were harvested from a healthy donor in accordance to the Dutch guidelines for secondary-use material, following established protocols.[1] HVSCs were cultured and expanded in advanced Dulbecco's modified Eagle's culture medium (a-DMEM; Gibco), supplemented with 10% FBS, 1% GlutaMax (Gibco), and 1% P/S. Cells were passaged at 80% confluency and seeded at passage 6 at a concentration of 8000 cell/cm^2 in 12-well

cell culture plates (Costar). Cells were cultured for an additional two days and subsequently exposed to 500 µL cytokine enriched medium from each 3D macrophage-scaffold culture at day 8 supplemented with 500 µL of fresh culture medium (a-DMEM, 10% FBS, 1% GlutaMax, 1% P/S). After 24 hrs all samples were washed with PBS (2 x for 5 seconds) and sacrificed for qPCR analysis (Tab.S1) as described in Sec. 2.6.4 for the 2D samples.

Supplementary Tab.S1. Primers for gene expression analysis of HVSCs

Primer	Symbol	Accession number	Primer Sequence (‘5-‘3)
α smooth muscle actin	αSMA	NM_001613.1	FW: CGTGTTGCCCCTGAAGAGCAT<br>RV: ACCGCCTGGATAGCCACATACA
Vimentin	VIM	NM_003380	FW: AAGACCTGCTCAATGTTAAGATC<br>RV: CTGCTCTCCTCGCCTTCC
Collagen type I	COL1A1	NM_000088	FW: AATCACCTGCGTACAGAACGG<br>RV: TCGTCACAGATCACGTCATCG
Collagen type III	COL3A1	NM_000090	FW: ATCTTGGTCAGTCCTATGC<br>RV: TGGAATTTCTGGGTTGGG
Elastin	ELN	NM_000501.3	FW: CTGGAATTGGAGGCATCG<br>RV: TCCTGGGACACCAACTAC
Transforming growth factor beta 1	TGFB1	NM_000660	FW: GCAACAATTCCTGGCGATACCTC<br>RV: AGTTCTTCTCCGTGGAGCTGAAG
Matrix Metalloproteinase 1	MMP1	NM_001145938.1	FW: CGCACAAATCCCTTCTACCC<br>RV: CTGTCGGCAAATTCGTAAGC
Matrix Metalloproteinase 2	MMP2	NM_001127891	FW: ATGACAGCTGCACCACTGAG<br>RV: ATTTGTTGCCCAGGAAAGTG
Metallopeptidase inhibitor 1	TIMP1	NM_003254.2	FW: TGACATCCGGTTCGTCTACA<br>RV: TGCAGTTTTCCAGCAATGAG

Exposing HVSCs to the conditioned media of the macrophages cultured on the various microarchitectures led to distinct gene expression profiles of the HVSCs after 24 hrs of culture (Fig.S6A). Interestingly, HVSCs exposed to the supernatant of macrophages cultured on 6 µm Ø anisotropic scaffolds showed a significant upregulation of the αSMA and COL3A1 genes, and significant downregulation of MMP1, when compared to HVSCs exposed to the supernatant of macrophages cultured on the other scaffold microarchitectures. The gene expression of VIM, COL1A1, TIMP1 and MMP2 was comparable for all groups.

HVSCs exposed to the supernatant of macrophages which were biochemically stimulated showed similar expression of VIM, αSMA, TGFB1 and TIMP1. However, HVSCs exposed to the supernatant of IFN-γ/LPS-treated macrophages showed significant downregulation of COL1A1 and COL3A1 and upregulation of MMP1. Exposure to the supernatant of IL-4/IL-13 treated macrophages resulted in significantly lower expression of ELN (Fig.6B).

Reference

1. Schnell AM, Hoerstrup SP, Zund G, et al. Optimal cell source for cardiovascular tissue engineering: Venous vs. aortic human myofibroblasts. *Thorac Cardiovasc Surg*. 2001;49(4):221-225. doi:10.1055/s-2001-16113
